# Super‐resolution for upper abdominal MRI: Acquisition and post‐processing protocol optimization using brain MRI control data and expert reader validation

**DOI:** 10.1002/mrm.27852

**Published:** 2019-07-01

**Authors:** Michael Ebner, Premal A. Patel, David Atkinson, Lucy Caselton, Louisa Firmin, Zahir Amin, Alan Bainbridge, Paolo De Coppi, Stuart A. Taylor, Sébastien Ourselin, Manil D. Chouhan, Tom Vercauteren

**Affiliations:** ^1^ Wellcome/EPSRC Centre for Interventional and Surgical Sciences University College London (UCL) London United Kingdom; ^2^ School of Biomedical Engineering and Imaging Sciences King's College London London United Kingdom; ^3^ Centre for Medical Imaging UCL London United Kingdom; ^4^ Department of Medical Physics and Biomedical Engineering University College London Hospitals NHS Foundation Trust London United Kingdom; ^5^ Institute of Child Health UCL London United Kingdom

**Keywords:** 3D reconstruction, MRCP, slice‐to‐volume registration, super resolution

## Abstract

**Purpose:**

Magnetic resonance (MR) cholangiopancreatography (MRCP) is an established specialist method for imaging the upper abdomen and biliary/pancreatic ducts. Due to limitations of either MR image contrast or low through‐plane resolution, patients may require further evaluation with contrast‐enhanced computed tomography (CT) images. However, CT fails to offer the high tissue‐ductal‐vessel contrast‐to‐noise ratio available on T2‐weighted MR imaging.

**Methods:**

MR super‐resolution reconstruction (SRR) frameworks have the potential to provide high‐resolution visualizations from multiple low through‐plane resolution single‐shot T2‐weighted (SST2W) images as currently used during MRCP studies. Here, we (i) optimize the source image acquisition protocols by establishing the ideal number and orientation of SST2W series for MRCP SRR generation, (ii) optimize post‐processing protocols for two motion correction candidate frameworks for MRCP SRR, and (iii) perform an extensive validation of the overall potential of upper abdominal SRR, using four expert readers with subspeciality interest in hepato‐pancreatico‐biliary imaging.

**Results:**

Obtained SRRs show demonstrable advantages over traditional SST2W MRCP data in terms of anatomical clarity and subjective radiologists’ preference scores for a range of anatomical regions that are especially critical for the management of cancer patients.

**Conclusions:**

Our results underline the potential of using SRR alongside traditional MRCP data for improved clinical diagnosis.

## 1 INTRODUCTION

Magnetic resonance (MR) cholangiopancreatography (MRCP) is an established method for imaging the upper abdomen and biliary/pancreatic ducts. Heavily T2‐weighted (HT2W) sequences exploit slow moving fluid in the biliary and pancreatic ducts to generate high‐resolution images of the biliary tree.^1^ Such HT2W images are acquired as near‐isotropic voxel three‐dimensional (3D) image volumes during free breathing using respiratory triggers and are useful in the assessment of intra‐ductal benign and malignant pathology.^2^, ^3^, ^4^ However, the assessment of peri‐ductal and extra‐ductal upper abdominal soft tissue pathology is reliant on traditional two‐dimensional (2D) single‐shot T2‐weighted (SST2W) imaging (e.g. half‐Fourier acquisition single‐shot turbo spin echo, HASTE sequences) because of the more suitable tissue contrast they provide of surrounding anatomy. To achieve acceptable in‐plane signal‐to‐noise ratio (SNR), slice thickness is increased, with resultant low through‐plane resolution, anisotropic voxel 2D images. Because of the close proximity of fine ductal/vascular structures in the upper abdomen, these 2D images are particularly susceptible to partial voluming effects (PVEs), whereby signal from a single voxel is contaminated by signal from multiple anatomical structures. Images are also obtained in breath‐hold, so that patient non‐compliance and breath‐hold difficulties commonly introduce inter‐slice motion artifact. To mitigate these effects, SST2W sequences can be obtained consecutively in axial and coronal planes, with radiologists reading low through‐plane resolution, motion‐artifacted image series in both planes to improve sensitivity to pathology. However, early malignant lesions are typically small and mural/extra‐ductal (rather than intra‐ductal) and easily overlooked. Diagnostic pathways are therefore reliant on non‐MR imaging modalities for the exclusion of small volume pathology, but these can be invasive (e.g. endoscopic ultrasound, EUS) or require ionizing radiation (e.g. computed tomography, CT).

*Super‐resolution reconstruction* (SRR) is a post‐processing technique to combine multiple low‐resolution (LR) 2D image stacks into a single high‐resolution (HR), 3D visualization. Applications of SRR in MR imaging (MRI) range from adult studies on the tongue^5^ and thorax^6^ to fetal applications.^7^, ^8^, ^9^ Despite being well‐suited to overcome the limitations of multiplanar SST2W in principle, its application in the upper abdomen to‐date has been limited. In fact, super‐resolution (SR) can only work accurately in case of very precise motion estimation with subvoxel accuracy for all LR observations for the recovery of subvoxel detail.^10^, ^11^, ^12^ This is especially difficult in the context of abdominal imaging where images acquired from separate breath‐holds are subject to inspiratory/expiratory variation in addition to deformation arising from cardiac motion, arterial pulsation, and gastro‐intestinal tract peristaltic motion. Existing respiratory motion models for motion correction require the availability of respiratory surrogate data^13^ which are currently not available for MRCP studies. Using an SRR approach such as the iterative two‐step registration‐reconstruction approach used in fetal MRI,^8^, ^14^ applied to only two stacks, is prone to generate a strongly biased volume and the currently used rigid motion models might not be sufficient.

Our preliminary study^15^ demonstrated the feasibility of upper abdominal MRI SRRs generated from only two standard MRCP protocol axial and coronal SST2W series using HT2W volumes as a reference‐guide for in‐plane deformable slice‐to‐volume (S2V) registration/motion correction. but anatomical clarity was lacking and a more robust registration/motion correction was needed. SRRs generated from a larger number of LR 2D source series are known to increase the reconstruction quality^9^, ^16^ but acquisition of additional SST2W data comes at the expense of additional patient scanning time. Insight on the optimal orientation and number of input stacks for SRR is limited,^17^, ^18^, ^19^ especially in the upper abdomen. Using HT2W volumes as a reference to guide registration is attractive but motion artifact arising from extended acquisition times and inconsistent breathing commonly degrades HT2W image quality. More rapidly acquired, similar to SST2W tissue contrast T2‐weighted balanced fast field echo (BFFE) sequences may offer a more consistent alternative for reference‐guided registration, as may other more recently proposed non‐reference guided SRR registration methods.^7^ Finally, in order to objectively assess these factors, control studies using imaging free from significant variation in inter‐subject motion artifact and from which a robust non‐motion artifacted ground‐truth/reference standard can be generated for SRR comparison are required.

In this pilot study, we obtained healthy volunteer multiplanar SST2W stacks of the upper abdomen and the brain (“quasi‐static” control data, to remove the effect of upper abdominal motion artifact), with the overall objectives of (i) optimizing source image acquisition protocols by establishing the ideal number and orientation of SST2W series (so‐called “source data configuration”) for MRCP SRR generation; (ii) optimizing post‐processing protocols by defining the best approach to registration/motion correction for SRR in the upper abdomen; and (iii) validation of the overall potential of upper abdominal SRR, using expert readers to compare pre‐specified imaging features on the SRR with imaging obtained from standard SST2W MRCP protocols.

## 2 METHODS

### 2.1 Subjects and MRI scanning

Local ethics committee approval was obtained and all participants provided informed written consent. Volunteers were recruited via advertisement within the University College London campus and were eligible if (i) they had no MRI contraindication, (ii) were not taking any long‐term medication (excluding the oral contraceptive pill), and (iii) had no documented history of previous liver or gastrointestinal disease. The final cohort consisted of eight healthy volunteers (six male, mean age (28 ± 2) years, mean weight (72 ± 12) kg). Imaging was performed using a 3T scanner (Ingenia, Philips Healthcare, Best, Netherlands) with a 16 channel body coil (SENSE XL Torso, Philips Healthcare, Best, Netherlands) used for abdominal imaging and a 15 channel head coil (dStream HeadSpine, Philips Healthcare, Best, Netherlands) used for brain imaging.

### 2.2 Image acquisition protocols

#### 2.2.1 Upper abdominal imaging

**Figure 1:**
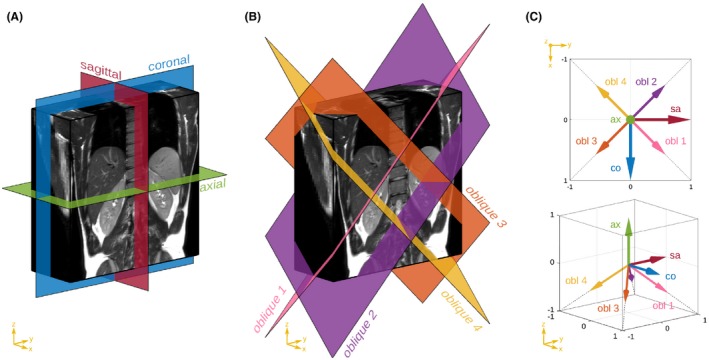
Visualization of acquisition planes of SST2W images. Left and middle figures illustrate the imaging planes in the standard and the oblique orientations, respectively. The associated slice‐select directions orthogonal to the respective acquisition planes are shown in the figure on the right. Example images are shown in Figure 2

**Figure 2:**
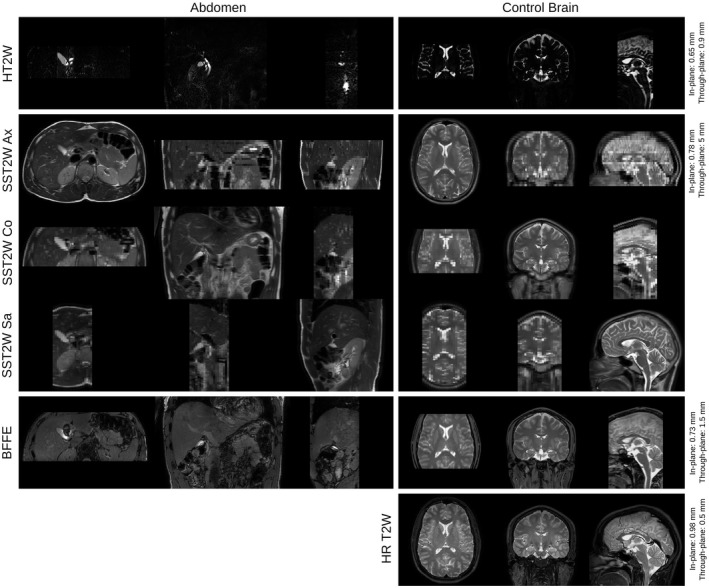
Images obtained by extended MRCP protocol for abdomen and brain anatomies. The first three rows show the acquisitions that are available in standard clinical MRCP studies, i.e. an axial and a coronal SST2W images and an HT2W volume. Further acquisitions include SST2W images in sagittal and oblique orientations (shown in Supporting Information Figure S1) and a BFFE volume as an alternative candidate for the reference‐guided motion correction framework. For validation purposes, a separate HR T2W volume was acquired for the brain

Abdominal imaging was planned to ensure adequate coverage of the liver and biliary tree, with acquisition parameters listed in Table 1. Standard clinical axial and coronal SST2W series were acquired in expiratory breath‐hold. The same acquisition parameters were used for additional expiratory breath‐hold SST2W series planned in (i) the sagittal plane, (ii) repeat axial, coronal and sagittal volumes shifted by half the slice thickness in the slice‐select direction, and (iii) four additional oblique volumes where the slice‐select dimensions were defined by the direction of a unit vector toward the lower four corners of a cube ${\left[-1,\;1\right]}^{3}$ whose orientation is aligned with the standard anatomical directions (Figures 1 and 2 and Supporting Information Figure S1).

**Table 1:** Image acquisition protocol used in this volunteer study for both abdominal and control brain anatomies

	Abdomen and Control Brain			Control Brain only
Description	HT2W volume	SST2W stack	BFFE volume	HR T2W volume
Acquisition Type	3D	2D	3D	3D
Repetition Time [ms]	1120	1161	2.46	2500
Echo Time [ms]	662.00	80.00	1.23	252.83
Flip Angle [${\;}^{\circ }$]	90	90	15	90
Pixel Spacing [mm]	0.65 × 0.65	0.78 × 0.78	0.73 × 0.73	0.98 × 0.98
Slice Thickness [mm]	1.8	5	1.5	1
Slice Spacing [mm]	0.9	5	1.5	0.5
Number of Slices	90	20‐25	83	360
Abdominal Imaging Trigger	Respiratory bellow	Expiratory BH	Expiratory BH	‐
Abdominal Scan Duration	04:06.0	00:21.5‐00:33.3	00:23.0	‐

*Notes*: For the abdominal imaging, the heavily T2‐weighted (HT2W) volume is acquired as a gated acquisition triggered by a respiratory bellow. The single‐shot T2‐weighted (SST2W) stack and the balanced fast field echo (BFFE) acquisitions are acquired at separate expiratory breath‐holds (BH). For the quasi‐static control brain experiment, no imaging trigger is used and an additional HR T2W volume is acquired for ground‐truth comparisons. Example images associated with this protocol are shown in Figure 2.

For deformable reference‐guided registration/motion correction, navigator‐triggered free‐breathing standard clinical HR HT2W volumes were acquired (Table 1). To investigate the potential of a less heavily T2‐weighted volume for reference‐guided registration that can be acquired more rapidly, we also used a thin slice 1.5 mm balanced fast field echo (BFFE) volume sequence to obtain high through‐plane resolution coronal images (Table 1).

#### 2.2.2 Quasi‐static control brain imaging

The same imaging protocol as presented for the abdomen was applied to the brain for seven of eight volunteers whereby identical imaging parameters were used to obtain image contrasts similar to the abdomen for the quasi‐static control brain studies. All control data was planned to ensure adequate coverage of the brain whereby no imaging trigger was used. For optimization studies and ground‐truth comparisons, an additional HR T2W volume was obtained (Table 1 and Figure 2).

### 2.3 Motion correction and volumetric reconstruction

Assuming a classical slice acquisition model^20^, ^21^ for each LR 2D SST2W slice acquisition $y_{s,\;i}\in R^{N_{s}}$ from a stack s∈S with slice index $i\in I_{s}$, the pixelwise association with the unknown HR volume $x\in R^{N}$, whereby $N_{s}\ll N$ for the voxel numbers due to the LR 2D image acquisition, can be expressed by

(1) $$y_{s,i}\left(j\right)=A_{s,i}\left(j,x\right)+n_{s,i}\left(j\right)\in R\;\text{for all slice voxels}\;j=1,\cdots ,N_{s}.$$

The linear operator $A_{s,\;i}\left(j,\;\cdot \right)$ acts as point spread function (PSF)‐defined intensity interpolator in the HR volume space that approximates the image acquisition process at a non‐linearly transformed physical position of voxel *j* of slice $y_{s,\;i}$ up to the imaging noise $n_{s,\;i}$. Each voxel intensity of a LR slice is therefore influenced by a certain neighborhood of this voxel within a HR volume **x** given by the assumed PSF that is specific to the slice profile of the LR MR acquisition.^22^ For SST2W sequences, a common approximation is given by a slice‐aligned 3D Gaussian function that depends on the in‐ and through‐plane resolution of the LR slice.^23^, ^24^ However, the motion that each anatomical region experiences during acquisition time is unknown. Once estimated, the HR volume can be obtained from the (motion corrected) LR slices by solving the associated SRR problem using a maximum a posteriori formulation (MAP)^12^, ^15^, ^20^

(2) $$x^{*}:=\underset{x\geq 0}{\text{argmin}}(\sum_{s\in S}\sum_{i\in I_{s}}\frac{1}{2}\|y_{s,i}-A_{s,i}{x\|}_{\ell^{2}}^{2}+\frac{\alpha }{2}{\left\|\nabla x\right\|}_{\ell^{2}}^{2})\in R^{N}$$

where $A_{s,\;i}x$ denotes the application of (1) to a vector in $R^{N_{s}}$, *α* ≥ 0 the regularization parameter and ∇ the differential operator. In particular, the linear operator $A_{s,\;i}:R^{N}\;\to \;R^{N_{s}},\;x\mapsto A_{s,\;i}x\;=:\;{\tilde{y}}_{s,\;i}$ models the image acquisition process that generates a LR slice ${\tilde{y}}_{s,\;i}$ from a HR volume **x** at a specific, and in our case, estimated, position and orientation within that HR volume. The second term of (2) is a first‐order Tikhonov regularization which corresponds to a MAP formulation exploiting a probabilistic prior on the HR volume. This counteracts the ill‐posed nature of the minimization problem and retains a computationally efficient least‐square structure. The final HR volume $x^{*}$ is also referred to as the SRR. More complex SRR models have been proposed in addition to the MAP formulation including modifying (2) to rely on robust M‐estimator^20^ and total variation formulations.^25^ However, while they substantially increase the computational cost, in our experience, they tend to show little improvement in the obtained reconstruction quality in case of appropriate motion correction. Associated experiments comparing Tikhonov and total variation regularizations are summarized in Supporting Information Tables S3 and S4 and Figures S6 and S7.

In this study, we evaluate two different motion correction methods: (i) a multimodal (With *multimodal* registration, we refer to the involvement of two images with different MR image contrasts due to different acquisition protocols as opposed to *monomodal* registration where two images of the same image contrast are used.) reference‐guided, in‐plane deformable registration approach that registers the LR SST2W slices to a separately acquired 3D HR reference volume of a different contrast (e.g. HT2W or BFFE volumes), and (ii) a monomodal rigid motion correction approach based on robust outlier rejection using only the SST2W slice image information.

#### 2.3.1 SRR using reference‐guided multimodal deformable motion correction

Building on our preliminary study,^15^ a reference‐guided motion correction approach is deployed whereby the following is assumed: (i) the resolution of the reference image is sufficiently high to act as a 3D reference volume, (ii) the occurring anatomical deformation can be captured by deforming the slice only in the in‐plane direction; the contribution in the orthogonal slice‐select direction can therefore be neglected given the thick slices and the associated intensity information uncertainty due to PVEs. Based on those assumptions the following non‐iterative three‐step volumetric reconstruction algorithm is performed: (i) multimodal slice‐to‐volume registration where each individual 2D slice of each stack is rigidly registered to the 3D reference; (ii) based on the intersection of the slices with the 3D reference, each slice is deformed in‐plane to compensate for non‐rigid deformations; and (iii) estimation of the SRR volume by solving (2) using the estimated deformations. Reference‐guided registration was applied using HT2W and BFFE volumes.

#### 2.3.2 Outlier‐robust SRR using monomodal rigid motion correction

Outlier‐robust SRR using rigid motion correction has been proposed recently for fetal MRI and exploits data from series obtained in at least three orientations.^8^, ^14^, ^20^, ^24^ Using an iterative two‐step registration‐reconstruction approach, a volumetric reconstruction step is followed by a rigid S2V registration step until convergence using the SST2W image series only. “Outlier” slices are detected during the SRR steps and rejected to prevent misregistered or artifact‐corrupted image slices from affecting the final SRR outcome. In this study, we deploy our recently presented method for fetal brain MRI.^7^ This computes the volumetric reconstruction using an SRR formulation similar to (2), i.e.

(3) $$x^{k+1}:=\underset{x\geq 0}{\text{argmin}}(\sum_{s\in S}\sum_{i\in I_{s,\sigma }^{k}}\frac{1}{2}\|y_{s,i}-A_{s,i}^{k}{x\|}_{\ell^{2}}^{2}+\frac{\alpha }{2}{\left\|\nabla x\right\|}_{\ell^{2}}^{2})$$

for a slice‐index set $I_{s,\;\sigma }^{k}\;:=\;\left\{i:\;\text{Sim}\left(y_{s,\;i},\;A_{s,\;i}^{k}x^{k-1}\right)\;\geq \;\sigma \right\}\;\subset \;I_{s}$ containing only slices that demonstrate high agreement with their simulated counterparts projected from the previous SRR iterate using a similarity measure Sim and parameter *σ* > 0. In particular, (2) is a special case of (3) since the combined linear forward operator $A_{s,\;i}^{k}$ describes the oriented PSF‐interpolator estimated after *k* motion correction steps, whereby only a subset of indices $I_{s,\;\sigma }^{k}\;\subset \;I_{s}$ per stack *s* is considered. Thus, (3) represents a convex SRR problem with complete outlier removal using a single hyperparameter *σ* in a linear least‐squares minimization formulation that can be solved efficiently using *matrix‐free* operations.^15^, ^26^

Therefore, using this iterative SRR framework it is assumed that, (i) sufficient input SST2W images are available to allow anatomically plausible reconstructions from these LR image stacks, (ii) the anatomical motion captured per slice is approximately rigid for the specified region of interest (ROI) for most of the slices, and (iii) the outlier‐rejection algorithm can reliably reject individual slices that present non‐matching deformations.

### 2.4 Data preprocessing and parametrization of the reconstruction pipeline

A ROI including the common bile duct, head of pancreas, porta hepatis, and central liver was specified manually using masks generated on axial SST2W images by a radiologist with over 10 years experience in abdominal imaging (PP). Quasi‐static control ROIs for the brain tissue were defined automatically using the Brain Extraction Tool (BET).^27^ This region was also used for the quantitative ground‐truth comparisons. SST2W images were preprocessed via an ITK bias field^28^ and a linear intensity correction step constrained by the provided manual (abdomen) and automatic (brain) masks.

For abdominal and quasi‐static control brain data, the ROI mask was propagated to all the remaining SST2W series using nearest neighbor interpolation. Reconstruction pipelines were developed in Python using itk for the individual registration steps. Only data within the masked ROI was used for image processing and all slice registrations were constrained to the slice mask. For the deformable, reference‐guided SRR framework, the in‐plane deformation was performed using NiftyReg (https://github.com/KCL-BMEIS/niftyreg) software that is based on a fast free‐deformation algorithm^29^ using localized normalized cross‐correlation (LNCC) as similarity measure. By applying the obtained in‐plane deformation to each individual slice $y_{s,\;i}$, the SRR problem (2) was solved using the transformed slices ${\left\{{\overset{˘}{y}}_{s,\;i}\right\}}_{s\in S,i\in I_{s}}$ in combination with the linear operators ${\overset{˘}{A}}_{s,\;i}\;=\;A_{s,\;i},\;s\in S,\;i\in I_{s},$ that carry the respective rigid slice motion correction estimates. To model the PSF of the image acquisition, we chose to approximate the SST2W sequence slice profile by a 3D Gaussian function defined by $\text{diag}(\frac{{\left(1.2\;s_{1}\right)}^{2}}{8\;\ln(2)},\;\frac{{\left(1.2\;s_{2}\right)}^{2}}{8\;\ln(2)},\;\frac{s_{3}^{2}}{8\;\ln(2)})\in R^{3\;\times \;3}$ as variance‐covariance matrix with $s_{1},\;s_{2}$ and $s_{3}$ representing the in‐ and through‐plane spacings^23^, ^24^ in the slice‐coordinate system, respectively. For the outlier‐robust, rigid motion correction and SRR framework, NiftyMIC (https://github.com/gift-surg/NiftyMIC) was used to solve (3) as described in Ebner et al.^7^ Three iterations of two‐step rigid S2V registration and outlier‐robust SRR steps were performed with normalized cross‐correlation (NCC) used to guide registrations. To create a first HR reference for the initial rigid S2V registrations, we used a discrete variant of Nadaraya‐Watson kernel regression as an efficient scattered data approximation scheme for the volumetrically aligned SST2W image stacks.^15^, ^30^ For outlier detection, Sim was set to NCC and *σ* was selected empirically with values of 0.6, 0.65 and 0.7 per iteration to account for increasing accuracy in (3), respectively. There is broad consensus that SR in MRI can only reliably be achieved in through‐plane and not in in‐plane direction.^12^, ^31^, ^32^, ^33^ We, therefore, defined the isotropic reconstruction grid for the abdominal SRRs by the in‐plane resolution of the stacks (0.78 mm). Given the SST2W slice thickness of 5 mm, our algorithm created an SR volume with approximately six times the resolution in the through‐plane direction as the source SST2W images. For the brain, an isotropic reconstruction grid of 0.98 mm was used to approximately match the HR T2W volume resolution for the quantitative comparisons. The regularization parameter *α* was set to be 0.01 and 0.02 for the abdominal and quasi‐static control SRRs, respectively. The different values are a result of the different reconstruction grid resolutions and were determined using a combination of L‐curve studies^34^ and visual inspections.

### 2.5 Evaluation methodology

#### 2.5.1 Optimization control studies for brain MRI SRR

Six source data configurations for SRR generation were evaluated, using (i) axial and coronal (“a+c”, two series); (ii) axial, coronal, and sagittal (“a+c+s”, three series); (iii) axial, coronal, sagittal, and slice‐select direction shifted axial, coronal, and sagittal (“2a+2c+2s”, six series); (iv) axial, coronal, sagittal, and the first three oblique planes as shown in Figure 1 (“a+c+s+3obl”, six series); (v) axial, coronal, sagittal, and all four oblique planes (“a+c+s+4obl”, seven series); and (vi) both axial, both coronal, both sagittal, and all four oblique planes (“2a+2c+2s+4obl”, ten series).

To evaluate the registration/motion correction approaches, five SRRs were generated for each source data configuration using (i) no registration/motion correction (static SRR); (ii) reference‐guided rigid registration using HT2W data (RG‐HT2W); (iii) reference‐guided rigid registration using BFFE data (RG‐BFFE); (iv) reference‐guided rigid registration using HR T2W data (RG‐HRT2W); and (v) outlier‐robust rejection rigid registration using only the SST2W source data used for each configuration (NiftyMIC). HR T2W brain imaging was used as ground‐truth/reference standard imaging, with NCC used for both the similarity measure for each of the registration steps and the quantification of ground‐truth similarity. Alternative similarity measures for registration (mutual information, MI; normalized mutual information, NMI) and ground‐truth similarity (structural similarity index measure,^35^ SSIM; NMI; peak‐signal‐to‐noise ratio, PSNR) were also generated and are presented in the Supporting Information Figures S3 and S4 and Table S1 for the sake of manuscript conciseness. Analysis of RG‐HRT2W based SRR results from the quasi‐static control data is used to establish the optimal number and orientation of SST2W series for SRR while excluding the confounding factor of motion as much as possible.

#### 2.5.2 Optimization studies for upper abdominal MRI SRR

The results obtained from the control brain studies were used to inform the abdominal imaging optimization study methodology. Three source data configurations for SRR generation were evaluated: (i) axial and coronal (“a+c”, two series), (ii) axial, coronal, and sagittal (“a+c+s”, three series), and (iii) axial, coronal, sagittal, and the first three oblique planes (“a+c+s+3obl”, six series). Four SRR approaches were evaluated for all previously utilized methods excluding the unavailable RG‐HRT2W approach in this scenario.

In the absence of ground‐truth/reference standard imaging, assessment was based on (i) numerical SRR self‐consistency similarity measures, and (ii) subjective semi‐quantitative analysis by two radiologists. Self‐consistency was defined as the similarity between each slice $y_{s,\;i}$ and its projected SRR counterpart $A_{s,\;i}x$ according to a similarity metric Sim, i.e. $\text{Sim}(y_{s,\;i},\;A_{s,\;i}x)$, whereby NCC was used as Sim (SSIM, NMI and PSNR are additionally presented in Supporting Information Figure S5 and Table S2).

Subjective semi‐quantitative evaluation was undertaken independently by two radiologists with over 10 years experience in abdominal imaging (MC, PP) blinded to the SRR source data configuration or registration/motion correction approach. The clarity of high signal intensity anatomical (biliary ductal) structures and presence of misregistration artifacts were scored as previously described (Ebner et al^15^; summarized in Supporting Information Section 2), and within‐subject SRRs were ranked in order of preference. Where inter‐reader discrepancies were noted, images were jointly re‐evaluated and a consensus score was recorded after joint re‐evaluation.

Because a reliable evaluation of 96 SRRs was not feasible, assessment was restricted to 24 SRRs at a time. The first experiment evaluated the three best performing image registration/motion correction approaches determined from numerical self‐consistency measures, in SRRs generated from the densest source data configuration (a+c+s+3obl, six series). The second experiment evaluated all three source data configurations for SRRs generated using the best‐performing MRCP SRR approach determined from the first experiment.

#### 2.5.3 Upper abdominal MRI SRR expert reader validation studies

Using the previously determined ideal number and orientation of SST2W series and the best approach for abdominal MRCP SRR, four radiologists (MC, PP, LF, and ZA), three with subspeciality interest in hepato‐pancreatico‐biliary imaging and all with more than eight years experience in abdominal imaging, independently validated upper abdominal SRRs by direct comparison with standard protocol axial and coronal SST2W images, using a semi‐quantitative scoring system. Both SRRs and standard SST2W images were scored for preservation of anatomical information at nine anatomical sites, focused predominantly on the assessment of peri‐ductal and extra‐ductal soft tissues. Readers also scored regions for the presence of artifacts, i.e. subjective but clinically apparent loss, addition or distortion of structures, introduced by SRR and recorded their subjective preference relative to standard SST2W images.

#### 2.5.4 Statistical analysis

Non‐parametric statistical tests were used for the non‐normally distributed NCC scores obtained from similarity measures between quasi‐static control SRRs and ground‐trust/reference standard imaging and for upper abdominal SRR self‐consistency. This included Wilcoxon signed‐rank tests for the quasi‐static control brain and Kruskal‐Wallis with post hoc Dunn's tests for the abdominal optimization studies. For the abdominal expert reader experiments, non‐parametric statistical tests were used for all reader‐derived semi‐quantitative scores, specifically Kruskal‐Wallis tests with post hoc Dunn's tests were used to determine differences between source data configurations or registration/motion correction strategies for upper abdominal SRRs. For validation studies, Bland‐Altman analysis of agreement for averaged clarity of anatomical information scores for SRR and standard SST2W imaging were compared using the median difference as a bias measure and 2.5th and 97.5th percentiles as the 95% Limits‐of‐Agreement (LoA). Wilcoxon signed‐rank tests were used to test for differences of anatomical clarity between SRRs and standard clinical axial and coronal SST2W data (“Ax&Co”), subjective preference and the presence of visible artifacts. The threshold of statistical significance for all tests was defined as *p* < 0.05.

## 3 RESULTS

### 3.1 Optimization control studies for brain MRI SRR

**Figure 3:**
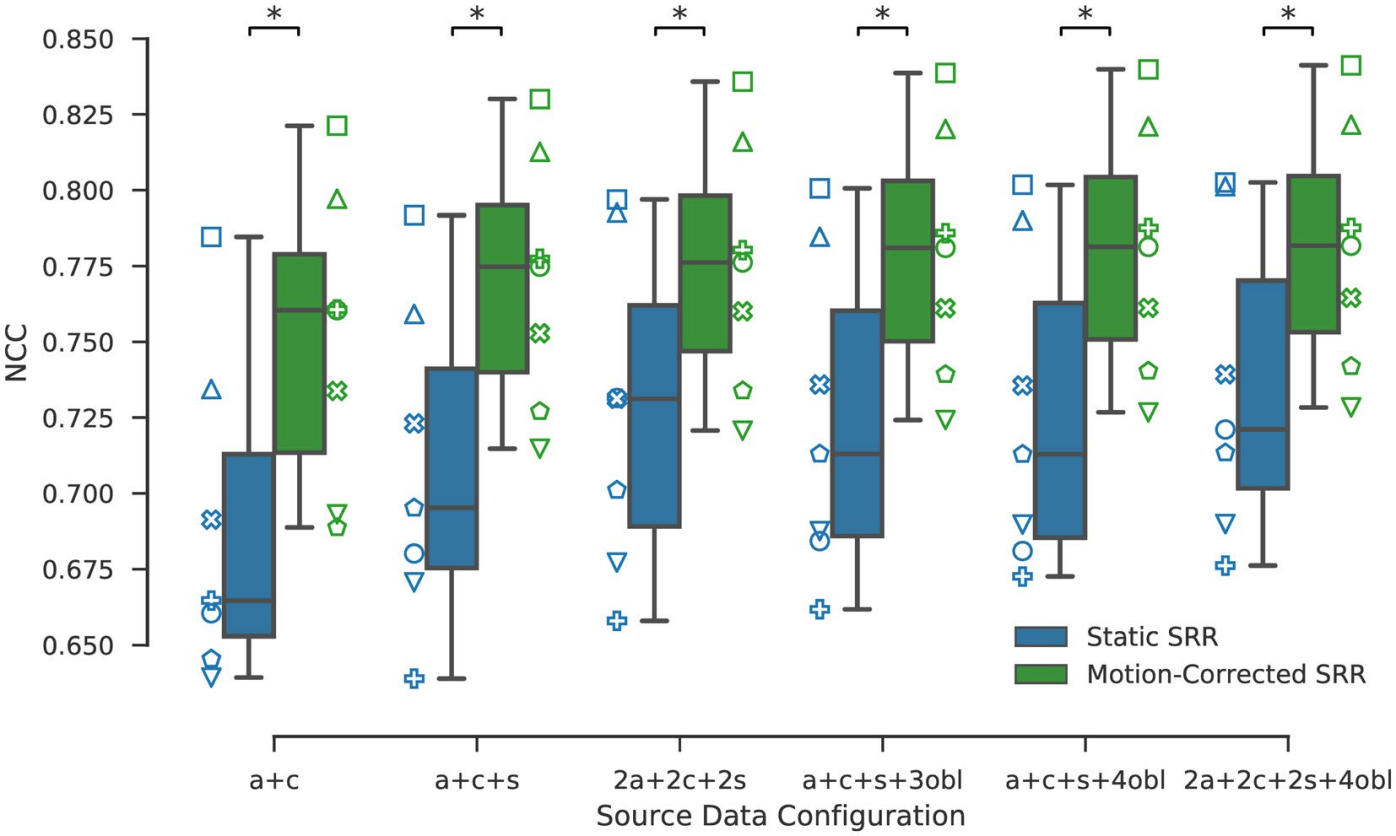
Ground‐truth (HR T2W) similarities for static and reference‐guided SRR outcomes for the quasi‐static control brain experiment whereby each of the seven subjects is assigned a different marker. The more input stacks are used the higher the similarity scores. Moreover, motion correction markedly improves the ground‐truth similarities which was performed by rigidly registering each individual slice to the HR T2W volume using NCC as the similarity measure. A visual comparison for one subject is provided in Figure 4. Stars indicate statistical differences between the groups using a pairwise Wilcoxon signed‐rank test (*p* < 0.05)

A total of 210 quasi‐static control brain SRRs were generated (30 SRRs for each of the seven volunteers). The box‐plot in Figure 3 illustrates the impact of motion correction and source data configuration on the NCC score. It shows that adding more than six input stacks (a+c+s+3obl) leads to only little numerical improvement in reconstruction quality for MRCP SST2W data (comparison using other similarity measures can be found in the Supporting Information Figures S3 and S4).

**Figure 4:**
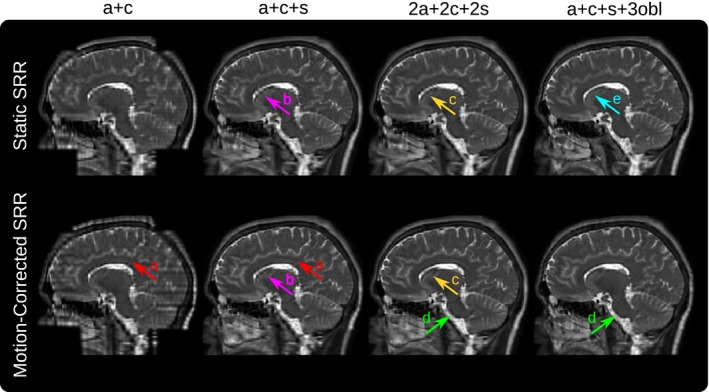
Qualitative comparison of the static and reference‐guided SRR outcome of one subject for various input data scenarios in the sagittal view (additional axial and coronal view comparisons are shown in Supporting Information Figure S2). It illustrates the impact of the number of input stacks and how multiple orientations can improve PVE recovery. In particular, SRR (a+c+s+3obl) shows visually higher anatomical accuracy than SRR (2a+2c+2s) despite the same number of six input stacks used for the SRR. The red arrows (A) underline that the SRR based on only two stacks (a+c) as currently available for clinical MRCP study protocols produces a very poor SRR quality which is especially noticeable in the sagittal view. The magenta arrows (B) illustrate that for three input stacks (a+c+s) the corpus callosum can only be reconstructed with limited geometrical integrity. Motion correction helps to recover it more clearly by adding three additional stacks (2a+2c+2s) as indicated by arrows (C). The green arrows (D) show the improved visual clarity at the medulla due to better PVE correction using oblique data. Additional oversampling for high input stack numbers leads to higher PSNR. This may also result in clear tissue boundaries even in case of insufficient motion correction for the static SRR as indicated by the cyan arrow (E)

A visual comparison in Figure 4 illustrates how different source data configurations affect the SRR results. In particular, using two input stacks (a+c) leads to inferior outcomes which is especially noticeable in the sagittal plane. Three input stacks (a+c+s) yield a substantial visual improvement that is further visible by adding three more stacks as shown for both 2a+2c+2s and a+c+s+3obl SRR outcomes. Although relatively subtle in this comparison, using oblique orientations (a+c+s+3obl) instead of same‐plane acquisitions (2a+2c+2s) can lead to more accurate SRRs depending on the curvature of tissue structures. This is indicated at the medulla which appears more blurred for the 2a+2c+2s outcome. Adding more stacks shows little visual improvement. However, the additional oversampling leads to higher PSNR and may result in clearer tissue boundaries (Figure 4 and Supporting Information Figure S2).

A quantitative comparison of the five registration/motion correction approaches with respect to different source data configurations is shown in Table 2. Only a subset of all performed comparisons is provided for simplicity (further comparisons are available in Supporting Information Table S1). The RG‐HRT2W outcome represents an approximation of the upper bound for the theoretically achievable MRCP SRR quality. Of the remaining four SRR approaches, using the BFFE volume as a reference performs second best. NiftyMIC, which does not rely on any external reference, performs between RG‐BFFE and RG‐HT2W. NiftyMIC demonstrated negligible outlier rejections (average 0.05 ± 0.21 slices) during SRR. In terms of source data configurations, numerical outcomes confirm the importance of multiplanar image input for high SRR quality. In particular, oblique planes (a+c+s+3obl) are preferable over multiple standard planes (2a+2c+2s).

**Table 2:** Ground‐truth (HR T2W) NCC‐similarities of obtained quasi‐static control brain SRRs for an increasing number of input stacks for different motion correction (MC) strategies summarized for all seven subjects

MC Strategy	a+c	a+c+s	2a+2c+2s	a+c+s+3obl
RG‐HRT2W	0.751 ± 0.046	0.770 ± 0.039	0.775 ± 0.038	0.779 ± 0.038
RG‐BFFE	0.735 ± 0.047	0.754 ± 0.039	0.759 ± 0.038	0.764 ± 0.038
NiftyMIC	0.724 ± 0.052	0.748 ± 0.043	0.751 ± 0.041	0.758 ± 0.040
RG‐HT2W	0.708 ± 0.042	0.734 ± 0.037	0.739 ± 0.037	0.750 ± 0.037
Static SRR	0.689 ± 0.049	0.708 ± 0.049	0.727 ± 0.050	0.724 ± 0.049

*Note*: The rows are sorted in a descending order according to the SRR outcome for “a+c+s+3obl”.

### 3.2 Optimization Studies for Upper Abdominal MRI SRR

**Figure 5:**
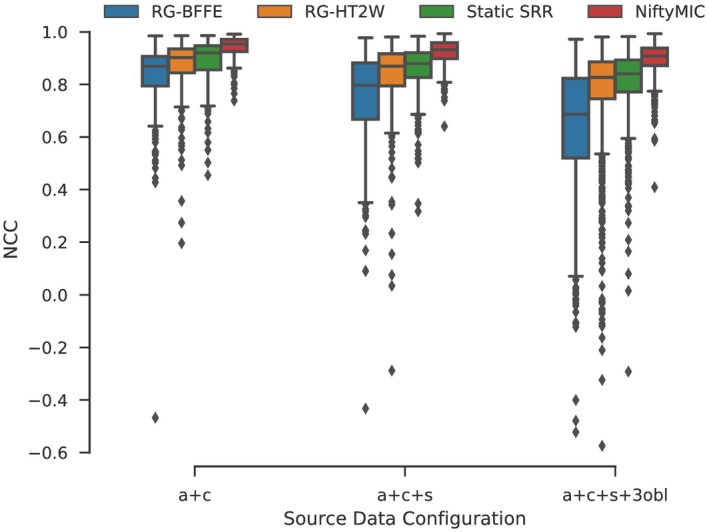
Self‐consistency evaluation given by projected NCC‐similarities for all slices of obtained abdominal SRRs for an increasing number of input stacks for different motion correction strategies summarized for all eight subjects. All self‐consistency outcomes between SRR approaches, except for RG‐HT2W vs Static SRR for “a+c+s,” are significantly different within each source data configuration based on Kruskal‐Wallis with post hoc Dunn tests (*p* < 0.05)

Based on the findings from the control quasi‐static brain MR data, we tested three source data configurations (a+c, a+c+s, a+c+s+3obl) using all four registration/motion correction methods available in the abdomen (RG‐BFFE, RG‐HT2W, NiftyMIC and static SRR). A total of 96 abdominal SRRs were generated (12 SRRs for each of the eight volunteers). The scores in Figure 5 indicate highest self‐consistency for the NiftyMIC SRR outcomes across source data configurations for NCC (more comparisons in Supporting Information Figure S5 and Table S2) followed by Static SRR. Lower outcomes for the reference‐guided approaches indicate the existence of slice misregistrations with RG‐HT2W producing consistently better results. For NiftyMIC, the statistics for the outlier‐robust framework were (# of rejected slices / # of total slices) 0.14 ± 0.35/30.43 ± 4.40, 0.71 ± 0.88/50.29 ± 4.33, and 5.00 ± 2.14/108.57 ± 4.87 for the a+c, a+c+s and a+c+s+3obl input data scenarios, respectively, indicating a moderate increase of slice rejections for increasing input data.

**Figure 6:**
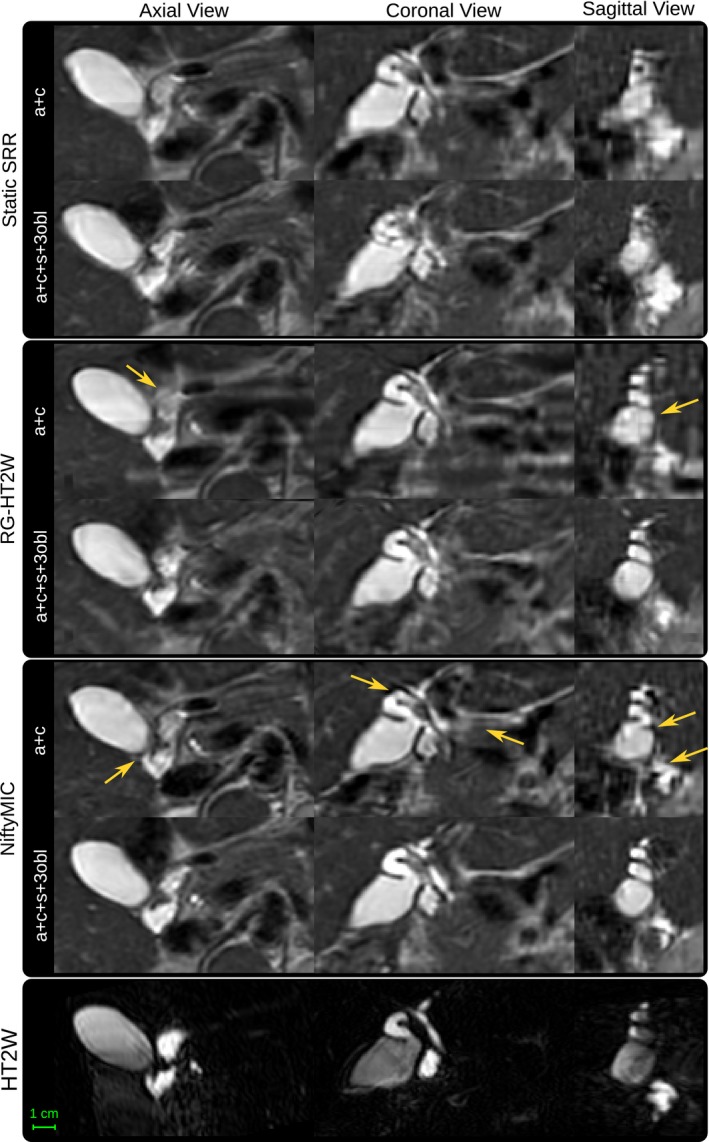
Qualitative comparison between the SRR approaches using either two or six input stacks. Both motion correction frameworks, i.e. the HT2W‐guided one and NiftyMIC, achieve SRRs with visually improved anatomical plausibility. However, in areas where MRCP lacks contrast, NiftyMIC tends to produce superior results. Moreover, using six input stacks can lead to better SRR outcomes in case of adequate motion correction which is especially visible in the sagittal view. Examples for such visual improvements are indicated by arrows

Figure 6 shows that SRRs based on fewer input stacks represent anatomically less plausible reconstructions. Both RG‐HT2W and NiftyMIC show improved anatomical clarity over the static SRR approach. This is especially the case for the SRRs based on six input stacks. However, RG‐HT2W becomes less accurate in areas with poor HT2W image contrast.

Table 3 summarizes the two radiologists’ qualitative evaluation. Superiority of NiftyMIC over RG‐HT2W and Static SRR in terms of anatomical clarity, amount of visible motion and the radiologists’ preference was statistically significant. By selecting NiftyMIC as the best‐performing MRCP SRR approach, a second experiment demonstrated the significantly better reconstruction quality achieved using the a+c+s+3obl source data. In particular, it was selected as the radiologists’ preference without exception. Two independently conducted experiments with, at least, days delay, show little variability in the radiologists’ assessment of the NiftyMIC a+c+s+3obl outcome, Table 3 rows 3 and 6.

**Table 3:** Clinical evaluation by two radiologists for abdomen averaged over all eight subjects for two separate experiments: A first experiment based on six input stacks (a+c+s+3obl), and a second experiment involving three source data combinations (a+c, a+c+s, and a+c+s+3obl) for the best performing method in the first experiment (NiftyMIC)

		Clarity of anatomical structures			Total Score Anatomical Clarity	Visible Motion	Radiologists’ Preference
		CBD	LHD	RHD			
Exp. 1: a+c+s+3obl	Static SRR	1.25 ± 0.46	1.25 ± 0.46	1.25 ± 0.46	3.75 ± 1.16	1.25 ± 0.46	1.62 ± 0.52
	RG‐HT2W	1.50 ± 0.76	1.62 ± 0.52	1.62 ± 0.52	4.75 ± 1.58	1.25 ± 0.71	1.38 ± 0.52
	NiftyMIC	2.88 ± 0.35	2.62 ± 0.52	2.62 ± 0.52	8.12 ± 1.25${\;}^{*}$	2.25 ± 0.71${\;}^{*}$	3.00 ± 0.00${\;}^{*}$
Exp. 2: NiftyMIC	a+c	1.38 ± 0.52	1.12 ± 0.35	1.12 ± 0.35	3.62 ± 0.74	1.00 ± 0.00	1.12 ± 0.35
	a+c+s	1.75 ± 0.46	1.50 ± 0.53	1.50 ± 0.53	4.75 ± 0.89	1.38 ± 0.52	1.88 ± 0.35
	a+c+s+3obl	2.62 ± 0.52	2.50 ± 0.76	2.25 ± 0.71	7.38 ± 1.41${\;}^{*}$	2.12 ± 0.35	3.00 ± 0.00${\;}^{*}$

*Notes*: Clarity of anatomical structures score indicates how well common bile duct (CBD), and left and right hepatic ducts (LHD and RHD) are visualized in each image with ratings 0 (structure not seen), 1 (poor depiction), 2 (suboptimal visualization; image not adequate for diagnostic purposes), 3 (clear visualization of structure but reduced tissue contrast; image‐based diagnosis feasible) and 4 (excellent depiction; optimal for diagnostic purposes). Visible motion score rates the amount of visible non‐corrected motion from score 0 (complete motion) to 3 (no motion). Radiologists’ preference ranks the subjectively preferred reconstructions from 1 to 3 (least to most preferred). Stars in the last three columns indicate that the respective score is statistically significantly different from the respective other two groups based on Kruskal‐Wallis and post hoc Dunn tests (*p* < 0.05).

### 3.3 Upper abdominal MRI SRR expert reader validation studies

**Figure 7:**
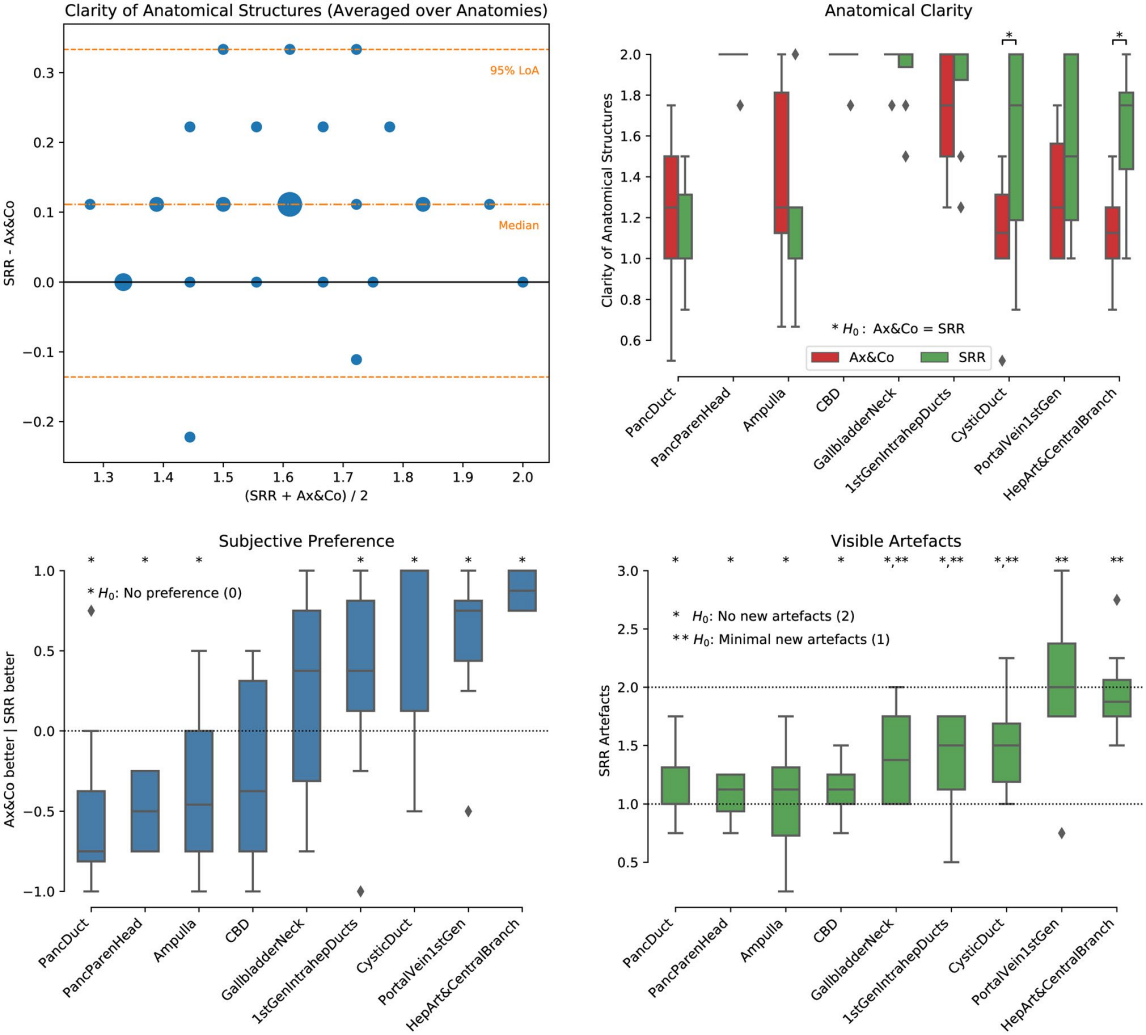
Clinical evaluation by four radiologists for the abdomen of the third experiment for all eight subjects. Top panels: Clinical interpretability scores were 0 (structure not identified), 1 (structure poorly visualized), and 2 (structure clearly visualized). Bottom left: Subjective impression score ranks how frequently the SRR (NiftyMIC a+c+s+3obl) was considered subjectively of worse, same or better quality than the original axial and coronal SST2W data for interpretation. Bottom right: Artifacts measure to what extent the SRR presented additional artifacts with respect to the original axial and coronal SST2W data with scores 0 (lots of new artifacts), 1 (minimal new artifacts), 2 (no new artifact), and 3 (less artifact than original). Stars are shown to illustrate significant outcomes by rejecting the respective $H_{0}$ hypothesis based on a Wilcoxon signed‐rank test (*p* < 0.05)

NiftyMIC with a+c+s+3obl input stacks was chosen as best‐performing method for subsequent validation studies. Bland‐Altman analysis of agreement between SRR and standard axial and coronal SST2W data in Figure 7 confirms a systematically better outcome in clarity of anatomical structures presented on the SRRs. Assessment of the individual anatomical regions shows a statistically better SRR performance for two of the nine assessed anatomical features (cystic duct, and hepatic artery and central branches). Expert reader subjective preference scores demonstrate statistically significant preferences for vascular structures, the cystic duct and first generation intrahepatic ducts. Pancreatic duct, head‐of‐pancreas parenchyma, and ampulla were preferred on standard imaging. Importantly, artifact scores were generally well above 1 which underlines that the SRRs present minimal or no new artifact in comparison with the original data. On average, no structure was scored as having less artifact than the original data.

## 4 DISCUSSION

In this work, we present the first comprehensive analysis of SRR for MRCP studies. Using quasi‐static control data from the brain and upper abdominal MR imaging of healthy volunteers, we optimized source data configuration and motion correction strategies for MRCP SRR. We validated optimized SRRs using expert readers to show that SRRs can lead to novel high‐quality HR visualization of peri‐ductal anatomy. In particular, we empirically showed that SRRs based on the clinically available axial and coronal images are of inferior quality compared to those with additional input stacks. By performing highly controlled brain experiments we found that after approximately six input stacks the SRR quality achievable with MRCP SST2W sequences plateaus. Moreover, we showed that not only the number but also the orientation of the SST2W stack acquisitions matters. In particular, for the same number of six input stacks using oblique orientations on top of the standard anatomical directions produces superior SRR outcomes compared to using multiple standard axial, coronal and sagittal anatomical acquisitions. Notably, motion correction was needed for these experiments to exclude the confounding factor of motion despite the ’static’ nature of brain imaging.

High anatomical fidelity of the SRRs relies on the accurate establishment of generally non‐linearly affected, anatomical correspondences captured by different SST2W stacks acquired at different breath‐holds. We explored two SRR frameworks that are based on different motion correction strategies: A non‐iterative framework based on reference‐guided multimodal, in‐plane deformable motion correction that leverages the existence of a separate HR volume of a different modality for motion correction, and an iterative outlier‐robust SRR framework that is based on monomodal rigid motion correction that does not rely on any external reference. For the reference‐guided approach, we found that the optimal combination of reference image and associated similarity measure for registration is difficult to determine reliably. Whereas the controlled quasi‐static brain experiments indicated high quality SRR outcomes using the BFFE combined with NCC, this set‐up failed in the abdominal experiments due to misregistrations. We hypothesize that this comes from more complex appearance differences between BFFE and SST2W image contrasts we noticed in the abdomen compared to the brain, including the more pronounced cancellation effects around fat‐water tissue boundaries typical for BFFE (Figure 2). Besides the reported similarity measures and references in here, we also ran experiments using NMI, MI, and LNCC as additional similarity measures and the standard axial SST2W series as alternative reference, all of which corroborated the findings using NCC (Supporting Information Tables S1 and S2). Among reference‐guided SRR frameworks applied to the abdomen, only the HT2W‐guided approach driven by NCC showed promising results in regions of high MRCP contrast but is prone to artifacts in areas where this not the case. Our results indicate that the RG‐HT2W‐based motion correction can lead to improved anatomical clarity compared to the Static SRR for the abdomen. However, it is prone to creating artifacts due to slice misregistrations which may degrade the overall reconstruction quality. Additional experiments suggest that the in‐plane deformation step does not substantially improve results (Supporting Information Table S2). Similarly, experiments incorporating the PSF for registration as described in Ebner et al^15^ remained inconclusive. Contrastingly, we found very encouraging outcomes for the outlier‐robust SRR approach that was originally developed for fetal MRI. Despite the use of a rigid motion correction model only, it could consistently generate SRRs of the biliary tree that have the potential for diagnostic use. We therefore conclude that the encountered motion was approximately rigid for the most part and that the outlier rejection mechanism is effective in eliminating slices where this was violated. In case of sufficient data oversampling, the SRR algorithm was then able to reconstruct anatomical structures with high anatomical clarity. Qualitative expert reader comparisons showed that the optimized SRRs tend to provide limited value for regions like ampulla, the head of pancreas parenchyma, the imaged pancreatic duct and the CBD where specifically developed MRCP SST2W sequences traditionally show high diagnostic yield. However, in regions where the SST2W data typically provides only limited insight such as the portal vein and first generation branches, hepatic artery and central branches, cystic duct, and the imaged first generation intrahepatic ducts the SRRs allow an assessment with much higher anatomical detail which has important applications particularly for the assessment of hepato‐pancreatico‐biliary cancers. Therefore, we believe that using SRRs alongside the original data has real potential to improve the diagnostic yield of standard MRCP imaging, and improve patient care by reducing delays introduced by the need for further investigations, particularly in the context of cancer care.

Limitations of this work include the analysis of a relatively small cohort of eight subjects. Moreover, this pilot study was designed based on healthy volunteers. Acquired data during breath‐holds of patients is expected to have more challenging motion artifacts. We therefore plan to apply the proposed extended MRCP protocol to a representative patient cohort to assess the clinical utility of MRCP SRR. Furthermore, we plan to make improvements on the reference‐guided framework. Using the BFFE as a reference appears promising in case a more robust similarity measure is available. In fact, recently proposed deep learning methods^36^, ^37^ could prove useful for this step or, as shown in other applications, be used to aid the motion correction as a whole.^38^ In this work, we performed manual segmentations to define the region of interest in the standard axial SST2W image for the SRR. This step could be automated similar to the work as proposed in, e.g.^7^ For NiftyMIC a unified motion correction/reconstruction framework could help to better constrain the registration steps that might also allow the incorporation of a deformable model. This could help achieve higher anatomical accuracy in correcting for the challenging deformations in the upper GI anatomy. Finally, validation with abdominal isotropic 3D sequences was not undertaken in this study, but remains subject of future work.

## Supporting information


**FIGURE S1** Images obtained by extended MRCP protocol for abdomen and brain anatomies
**FIGURE S2** Qualitative comparison of the static and reference‐guided SRR outcome of one subject for various input data scenarios
**FIGURE S3** Ground‐truth (HR T2W) similarities for static and reference‐guided SRR outcomes for the quasi‐static brain experiment involving seven subjects
**FIGURE S4** Ground‐truth (HR T2W) similarities for the quasi‐static brain experiment for all registration/motion correction strategies as an extension to Figure S3
**FIGURE S5** Projected slice similarity evaluation for all slices of obtained abdominal SRRs for an increasing number of input stacks for different motion correction strategies summarized for all eight subjects
**FIGURE S6** Ground‐truth (HR T2W) similarities for first‐order Tikhonov (TK1) and isotropic Total Variation (TV) regularization SRR outcomes for the quasi‐static brain experiment involving seven subjects
**FIGURE S7** Qualitative comparison of the impact of using either first‐order Tikhonov (TK1) and isotropic Total Variation (TV) regularization in the final reconstruction step using NiftyMIC (a+c+s+3obl)
**TABLE S1** Ground‐truth (HR T2W) similarities of obtained quasi‐static control brain SRRs for an increasing number of input stacks for different motion correction (MC) strategies summarized for all seven subjects
**TABLE S2** Projected slice similarity evaluation of obtained abdominal SRRs for an increasing number of input stacks for different motion correction strategies summarized for all eight subjects
**TABLE S3** Ground‐truth (HR T2W) similarities of obtained quasi‐static control brain SRRs using first‐order Tikhonov (TK1) and isotropic Total Variation (TV) regularization SRR outcomes for an increasing number of input stacks for all seven subjects
**TABLE S4** Typical computational times to create a HR visualization of the biliary tree split into motion correction and volumetric reconstruction processing times
