# Supplementary material for: Super‐resolution for upper abdominal MRI: Acquisition and post‐processing protocol optimization using brain MRI control data and expert reader validation
Source: Magn Reson Med. 2019 Jul 1;82(5):1905–19. doi: 10.1002/mrm.27852 (PMC6742507; doi:10.1002/mrm.27852)
Supplement: Supplementary file 1 — FIGURE S1 Images obtained by extended MRCP protocol for abdomen and brain anatomies FIGURE S2 Qualitative comparison of the static and reference‐guided SRR outcome of one subject for various input data scenarios FIGURE S3 Ground‐truth (HR T2W) similarities for static and reference‐guided SRR outcomes for the quasi‐static brain experiment involving seven subjects FIGURE S4 Ground‐truth (HR T2W) similarities for the quasi‐static brain experiment for all registration/motion correction strategies as an extension to Figure S3 FIGURE S5 Projected slice similarity evaluation for all slices of obtained abdominal SRRs for an increasing number of input stacks for different motion correction strategies summarized for all eight subjects FIGURE S6 Ground‐truth (HR T2W) similarities for first‐order Tikhonov (TK1) and isotropic Total Variation (TV) regularization SRR outcomes for the quasi‐static brain experiment involving seven subjects FIGURE S7 Qualitative comparison of the impact of using either first‐order Tikhonov (TK1) and isotropic Total Variation (TV) regularization in the final reconstruction step using NiftyMIC (a+c+s+3obl) TABLE S1 Ground‐truth (HR T2W) similarities of obtained quasi‐static control brain SRRs for an increasing number of input stacks for different motion correction (MC) strategies summarized for all seven subjects TABLE S2 Projected slice similarity evaluation of obtained abdominal SRRs for an increasing number of input stacks for different motion correction strategies summarized for all eight subjects TABLE S3 Ground‐truth (HR T2W) similarities of obtained quasi‐static control brain SRRs using first‐order Tikhonov (TK1) and isotropic Total Variation (TV) regularization SRR outcomes for an increasing number of input stacks for all seven subjects TABLE S4 Typical computational times to create a HR visualization of the biliary tree split into motion correction and volumetric reconstruction processing times [file MRM-82-1905-s001.pdf]

Supporting Information

Super-Resolution for Upper Abdominal MRI: Acquisition and Post-Processing Protocol Optimisation using Brain MRI Control Data and Expert-Reader Validation

Michael Ebner, Premal A Patel, David Atkinson, Lucy Caselton, Louisa Firmin, Zahir Amin, Alan Bainbridge, Paolo De Coppi, Stuart Taylor, Sébastien Ourselin, Manil Chouhan, Tom Vercauteren

| OPTIMISATION CONTROL STUDIES FOR BRAIN MRI SRR

Figures S3 and S4 show SSIM, PSNR and NMI in addition to NCC as provided in Main Manuscript Figure 3.

Table S1 provides an extension to Main Manuscript Table 2 for more source data configurations, additional similarity measures and the axial SST2W stack (SST2WAx) as another possible choice as reference image for the reference-guided SRR approach. Using the short-hand "RG-Reference-SimilarityMeasureForRegistration", the settings for the reference-guided SRR approach are shown, where, e.g., RG-BFFE-NMI refers to the use of BFFE as reference volume for guidance and NMI as similarity measure for registration. Using NMI, as shown for the two references of HT2W and the axial SST2W stack in Table S1, can be computationally unstable and eventually fail as voxel numbers can be insufficient for the slice-to-volume metric evaluations.

| OPTIMISATION STUDIES FOR UPPER ABDOMINAL MRI SRR

Figure S5 shows SSIM, PSNR and NMI in addition to NCC as provided in the main paper in Main Manuscript Figure 5. Table S2 provides a numerical summary of investigated motion-correction strategies including the reference-guided approaches without the in-plane deformation step (RigidOnly).

For the qualitative comparison as shown in the Main Manuscript Table 3, two radiologists, blinded to the reconstruction methods, individually assessed each reconstruction side-by-side. The final score is a joint agreement of the radiologists' individual results. Scores were given for:

1. Anatomical clarity: based on how well common bile duct (CBD), left and right hepatic duct (LHD & RHD) were visualized
2. Visible motion: based on the degree of visible motion artefacts
3. Radiologists' preferred reconstruction

| ABDOMINAL MRI SRR USING TOTAL VARIATION REGULARIZATION

To investigate a potential improvement of the volumetric reconstruction quality by using a different regularizer other than the proposed first-order Tikhonov regularization (TK1), we additionally performed comparisons against isotropic total variation (TV) regularization. Thus, we compared the obtained SR reconstructions

$$\mathbf{x}^* := \operatorname*{arg\,min}_{\mathbf{x}\geq 0} \Big(\sum_{s\in\mathcal{S}} \sum_{i\in\mathcal{I}_s} \frac{1}{2}\|\mathbf{y}_{s,i} - \mathbf{A}_{s,i}\mathbf{x}\|_{\ell^2}^2 + \alpha\,\Psi(\mathbf{x}) \Big) \in \mathbb{R}^N \tag{S1}$$

using both the TK1 and TV regularizers defined as

$$\Psi(\mathbf{x}) = \mathrm{TK}_1(\mathbf{x}) := \frac{1}{2}\|\nabla\mathbf{x}\|_{\ell^2}^2 := \frac{1}{2}\sum_{k=1}^{N} \left(\partial_x \mathbf{x}(k)\right)^2 + \left(\partial_y \mathbf{x}(k)\right)^2 + \left(\partial_z \mathbf{x}(k)\right)^2 \tag{S2}$$

and

$$\Psi(\mathbf{x}) = \mathrm{TV}_{\mathrm{iso}}(\mathbf{x}) := \||\nabla\mathbf{x}|\|_{\ell^1} := \sum_{k=1}^{N} \sqrt{\left(\partial_x \mathbf{x}(k)\right)^2 + \left(\partial_y \mathbf{x}(k)\right)^2 + \left(\partial_z \mathbf{x}(k)\right)^2}, \tag{S3}$$

respectively. However, while the TK1 problem can be solved efficiently using a linear least-squares formulation the TV formulation requires a more complex framework that can deal with the associated non-smooth (but still convex) optimisation problem. For the implementation of the isotropic TV solver we used a primal-dual (PD) algorithm presented in [1] known for its suitability and fast convergence in imaging problems. Our PD solver implementation is publicly available in the NSoL[1] package which is integrated into NiftyMIC.

To allow for a direct comparison of the TV and TK1 regularizer outcomes, their respective SRR problem (S1) was solved after performed motion correction of the respective MRCP SRR frameworks as presented in the Main Manuscript Sections 2.3 and 2.3 (SRR using reference-guided multimodal deformable motion correction and Outlier-robust SRR using monomodal rigid motion correction). Therefore, for the non-iterative reference-guided approach, (S1) was solved after finishing the individual slice registrations. For the iterative two-step registration-reconstruction framework NiftyMIC, the respective regularizer is applied before the final SRR reconstruction step. This process allows a direct comparison of the TV and TK1 regularizers on the obtained SRR outcome without the confounding factor of different motion correction estimates. Additionally, this helps to keep the computational times low for NiftyMIC as the TV problem only needs to be solved once at the end of the two-step iterations.

Similar to the parametrisation of the TK1-based reconstruction pipeline described in the Main Manuscript Section 2.4 (Data Preprocessing and Parametrisation of the Reconstruction Pipeline), parameter studies were performed to determine suitable TV-regularization parameters α and the number of required PD iterations to achieve convergence. Based on additional visual inspection, TV-regularization parameters of $\alpha \in \{0.0001, 0.0005, 0.0009\}$ were chosen for the comparisons in here. Considering the input source data configurations of 'a+c', 'a+c+s' and 'a+c+s+3obl', it was found that 15 PD iterations are sufficient to achieve convergence for the chosen regularization parameters.

Table S3 provides a direct comparison of the obtained ground-truth (HR T2W) similarities for the quasi-static control brain experiment using TK1 and TV regularization for the SRR after RG-HRT2W-NCC-based motion correction. A visual summary of the outcomes is also provided in Figure S6 comparing TK1 and TV using $\alpha = 0.0005$. The comparisons show that TV does not lead to an improvement of the SRR as quantified by the similarity measures of NCC, SSIM and NMI. However, TV shows slightly increased PSNR compared to TK1. A qualitative comparison in Figure S7 represents an extension to Main Manuscript Figure 6 and shows that TV produces visually similar reconstructions compared to TK1 for low regularization parameters α. Larger α values for TV can lead to slightly sharper contours but a delicate balance needs to be found in order to avoid the introduction of staircase artefacts typical for TV which may well suppress clinically relevant structural information.

Finally, typical computational times to reconstruct a HR volume around the biliary tree anatomy with our non-optimized implementation are shown in Table S4 as measured on a local workstation using 8 CPUs. For the SRR obtained by NiftyMIC using the 'a+c+s+3obl' as source data configuration results in a total computational time of about 40 min using a TK1 regularizer. Using TV instead increases the total computational time by nearly 300 % to about 2 h 30 min.

Overall, our results underline that TV regularization substantially increases the computational cost but tends to show only little improvement in the obtained reconstruction quality.

REFERENCES

[1] Chambolle A, Pock T. A First-Order Primal-Dual Algorithm for Convex Problems with Applications to Imaging. Journal of Mathematical Imaging and Vision 2011 may;40(1):120–145. http://link.springer.com/10.1007/s10851-010-0251-1.

[1] https://github.com/gift-surg/NSoL

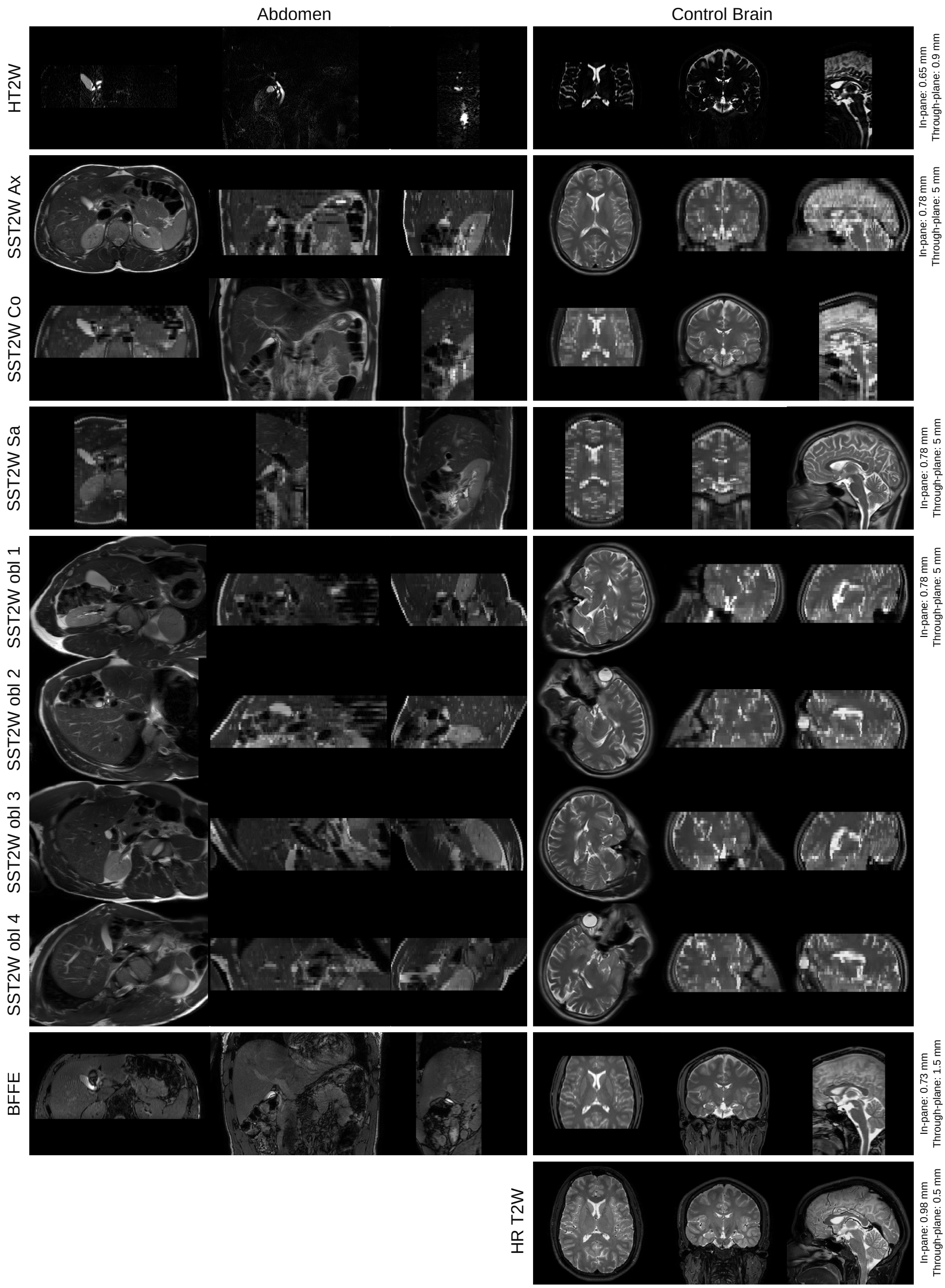

FIGURE S1 Images obtained by extended MRCP protocol for abdomen and brain anatomies. The first three rows show the acquisitions that are available in standard clinical MRCP studies, i.e. an axial and a coronal SST2W images and an HT2W volume. Further acquisitions include SST2W images in sagittal and oblique orientations and a BFFE volume as an alternative candidate for the reference-guided motion correction framework. For validation purposes, a separate HR T2W volume was acquired for the brain.

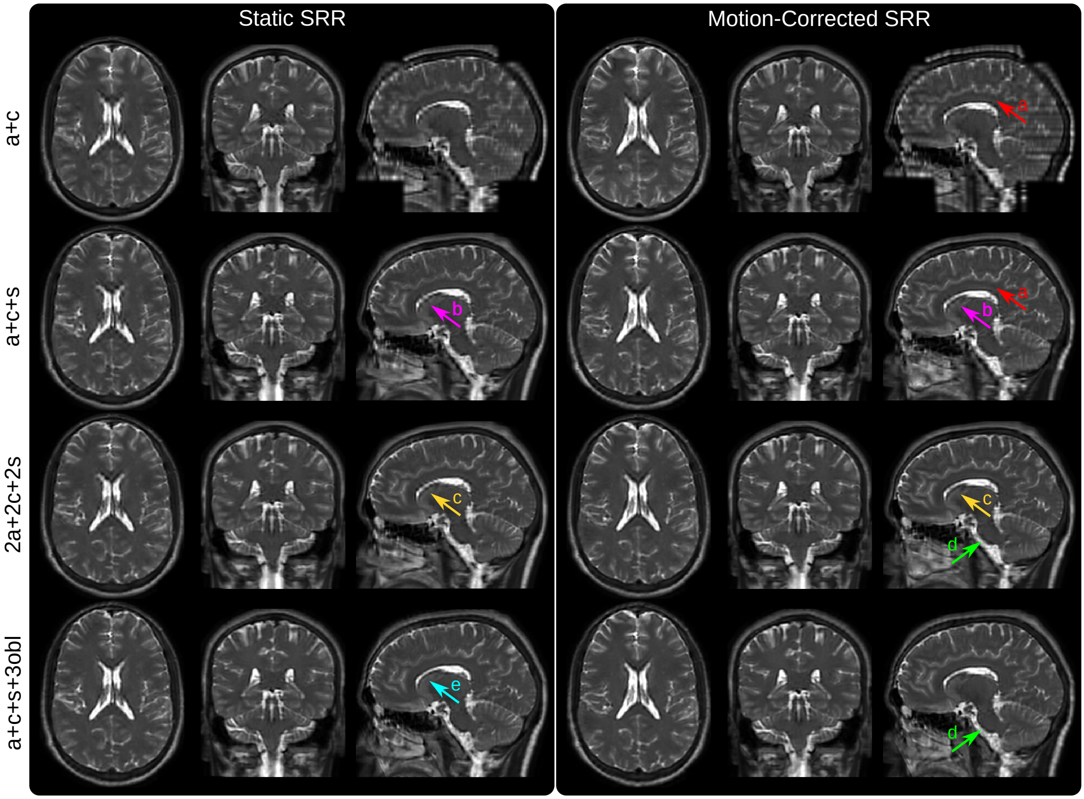

FIGURE S2 Qualitative comparison of the static and reference-guided SRR outcome of one subject for various input data scenarios. It illustrates the impact of the number of input stacks and how multiple orientations can improve PVE recovery. In particular, SRR (a+c+s+3obl) shows visually higher anatomical accuracy than SRR (2a+2c+2s) despite the same number of six input stacks used for the SRR. The red arrows (a) underline that the SRR based on only two stacks (a+c) as currently available for clinical MRCP study protocols produces a very poor SRR quality which is especially noticeable in the sagittal view. The magenta arrows (b) illustrate that for three input stacks (a+c+s) the corpus callosum can only be reconstructed with limited geometrical integrity. Motion-correction helps to recover it more clearly by adding three additional stacks (2a+2c+2s) as indicated by arrows (c). The green arrows (d) show the improved visual clarity at the medulla due to better PVE correction by using oblique data. Additional oversampling for high input stack numbers leads to higher PSNR. This may also result in clear tissue boundaries even in case of insufficient motion correction for the static SRR as indicated by the cyan arrow (e).

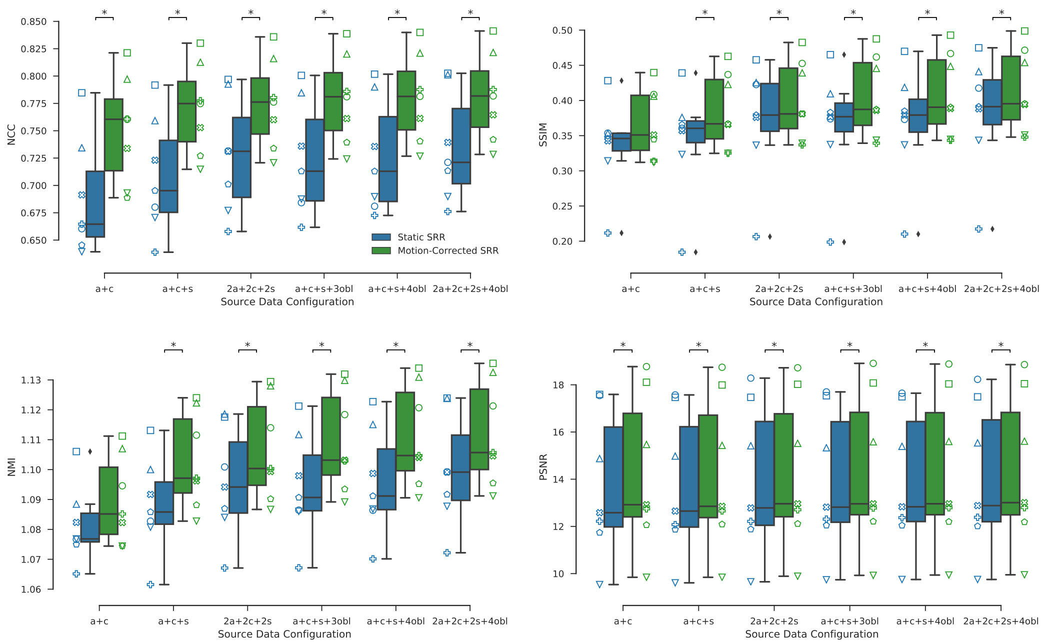

FIGURE S3 Ground-truth (HR T2W) similarities for static and reference-guided SRR outcomes for the quasi-static brain experiment involving seven subjects. The more input stacks are used the higher the similarity scores. Moreover, motion correction markedly improves the ground-truth similarities which was performed by rigidly registering each individual slice to the HR T2W volume using NCC as the similarity measure. Stars indicate statistical differences between the groups using a pairwise Wilcoxon signed-rank test ($p < 0.05$).

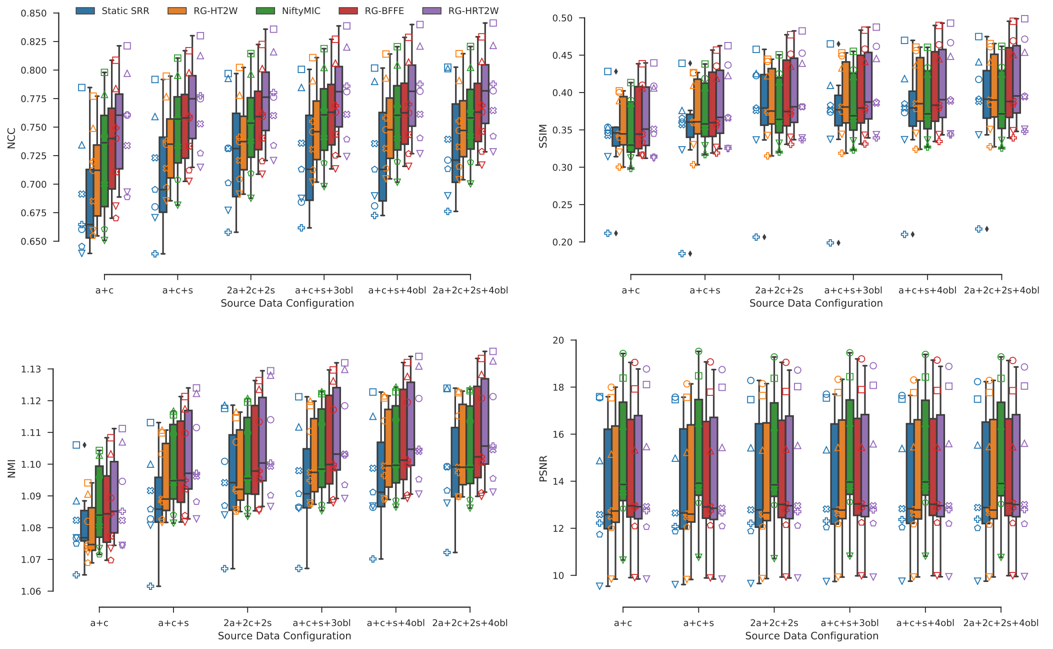

FIGURE S4 Ground-truth (HR T2W) similarities for the quasi-static brain experiment for all registration/motion correction strategies as an extension to Figure S3. Reference-guided approaches used NCC as the similarity measure for registration.

TABLE S1 Ground-truth (HR T2W) similarities of obtained quasi-static control brain SRRs for an increasing number of input stacks for different motion correction (MC) strategies summarized for all seven subjects. The rows are sorted in a descending order according to the SRR outcome for 'a+c+s+3obl'.

(a) NCC

MC Strategy	a+c	a+c+s	2a+2c+2s	a+c+s+3obl	a+c+s+4obl	2a+2a+2c+4obl
**RG-HRT2W-NCC**	0.751 ± 0.046	0.770 ± 0.039	0.775 ± 0.038	0.779 ± 0.038	0.780 ± 0.038	0.781 ± 0.038
**RG-HRT2W-MI**	0.722 ± 0.050	0.760 ± 0.040	0.772 ± 0.038	0.777 ± 0.038	0.780 ± 0.037	0.784 ± 0.037
**RG-HRT2W-NMI**	0.738 ± 0.051	0.760 ± 0.043	0.764 ± 0.042	0.771 ± 0.041	0.771 ± 0.041	0.772 ± 0.041
**RG-BFFE-NCC**	0.735 ± 0.047	0.754 ± 0.039	0.759 ± 0.038	0.764 ± 0.038	0.765 ± 0.038	0.766 ± 0.038
**RG-BFFE-NMI**	0.726 ± 0.052	0.748 ± 0.043	0.751 ± 0.042	0.758 ± 0.041	0.760 ± 0.041	0.759 ± 0.041
**NiftyMIC**	0.724 ± 0.052	0.748 ± 0.043	0.751 ± 0.041	0.758 ± 0.040	0.759 ± 0.040	0.758 ± 0.040
**RG-HT2W-NCC**	0.708 ± 0.042	0.734 ± 0.037	0.739 ± 0.037	0.750 ± 0.037	0.752 ± 0.037	0.751 ± 0.037
**RG-SST2WAx-NCC**	0.706 ± 0.049	0.717 ± 0.042	0.724 ± 0.041	0.735 ± 0.041	0.739 ± 0.040	0.739 ± 0.043
**Static SRR**	0.689 ± 0.049	0.708 ± 0.049	0.727 ± 0.050	0.724 ± 0.049	0.726 ± 0.048	0.735 ± 0.046
**RG-BFFE-MI**	0.353 ± 0.176	0.389 ± 0.196	0.427 ± 0.190	0.484 ± 0.183	0.506 ± 0.178	0.525 ± 0.168
**RG-SST2WAx-MI**	0.295 ± 0.052	0.336 ± 0.059	0.384 ± 0.053	0.414 ± 0.052	0.434 ± 0.048	0.463 ± 0.046
**RG-HT2W-MI**	0.242 ± 0.074	0.258 ± 0.079	0.310 ± 0.092	0.288 ± 0.094	0.290 ± 0.092	0.336 ± 0.101
**RG-SST2WAx-NMI**	–	–	–	–	–	–
**RG-HT2W-NMI**	–	–	–	–	–	–

(b) SSIM

MC Strategy	a+c	a+c+s	2a+2c+2s	a+c+s+3obl	a+c+s+4obl	2a+2a+2c+4obl
**RG-HRT2W-NMI**	0.378 ± 0.045	0.394 ± 0.050	0.408 ± 0.052	0.411 ± 0.054	0.414 ± 0.055	0.419 ± 0.055
**RG-HRT2W-NCC**	0.368 ± 0.047	0.386 ± 0.051	0.402 ± 0.053	0.407 ± 0.054	0.411 ± 0.055	0.416 ± 0.055
**RG-BFFE-NMI**	0.372 ± 0.046	0.386 ± 0.051	0.400 ± 0.054	0.404 ± 0.056	0.407 ± 0.057	0.412 ± 0.057
**RG-BFFE-NCC**	0.366 ± 0.047	0.382 ± 0.051	0.397 ± 0.053	0.402 ± 0.055	0.406 ± 0.056	0.411 ± 0.056
**RG-MRCP-NCC**	0.357 ± 0.038	0.374 ± 0.045	0.388 ± 0.046	0.393 ± 0.049	0.398 ± 0.050	0.403 ± 0.050
**RG-HRT2W-MI**	0.336 ± 0.055	0.365 ± 0.052	0.385 ± 0.052	0.391 ± 0.053	0.397 ± 0.053	0.405 ± 0.053
**NiftyMIC**	0.351 ± 0.041	0.374 ± 0.043	0.381 ± 0.046	0.385 ± 0.047	0.389 ± 0.047	0.389 ± 0.048
**RG-SST2WAx-NCC**	0.356 ± 0.043	0.358 ± 0.044	0.373 ± 0.047	0.382 ± 0.049	0.389 ± 0.050	0.388 ± 0.050
**Static SRR**	0.336 ± 0.060	0.344 ± 0.073	0.372 ± 0.077	0.364 ± 0.076	0.368 ± 0.075	0.382 ± 0.078
**RG-BFFE-MI**	0.099 ± 0.108	0.115 ± 0.130	0.129 ± 0.141	0.145 ± 0.151	0.154 ± 0.155	0.159 ± 0.157
**RG-SST2WAx-MI**	0.087 ± 0.022	0.086 ± 0.018	0.105 ± 0.018	0.101 ± 0.020	0.107 ± 0.021	0.121 ± 0.022
**RG-MRCP-MI**	0.059 ± 0.022	0.069 ± 0.022	0.084 ± 0.028	0.066 ± 0.026	0.065 ± 0.025	0.080 ± 0.028
**RG-SST2WAx-NMI**	–	–	–	–	–	–
**RG-MRCP-NMI**	–	–	–	–	–	–

(c) NMI

MC Strategy	a+c	a+c+s	2a+2c+2s	a+c+s+3obl	a+c+s+4obl	2a+2a+2c+4obl
**RG-HRT2W-NMI**	1.092 ± 0.013	1.104 ± 0.015	1.108 ± 0.016	1.111 ± 0.016	1.112 ± 0.016	1.113 ± 0.017
**RG-HRT2W-NCC**	1.090 ± 0.014	1.103 ± 0.015	1.107 ± 0.016	1.110 ± 0.016	1.111 ± 0.016	1.112 ± 0.016
**RG-BFFE-NMI**	1.087 ± 0.013	1.100 ± 0.015	1.104 ± 0.016	1.107 ± 0.016	1.109 ± 0.016	1.109 ± 0.017
**RG-BFFE-NCC**	1.087 ± 0.014	1.100 ± 0.015	1.104 ± 0.016	1.107 ± 0.016	1.109 ± 0.016	1.109 ± 0.017
**RG-HRT2W-MI**	1.078 ± 0.015	1.096 ± 0.015	1.101 ± 0.016	1.104 ± 0.015	1.107 ± 0.016	1.108 ± 0.016
**NiftyMIC**	1.088 ± 0.013	1.099 ± 0.013	1.102 ± 0.014	1.104 ± 0.014	1.105 ± 0.014	1.105 ± 0.014
**RG-MRCP-NCC**	1.079 ± 0.009	1.095 ± 0.012	1.099 ± 0.012	1.102 ± 0.013	1.104 ± 0.013	1.105 ± 0.014
**RG-SST2WAx-NCC**	1.087 ± 0.012	1.091 ± 0.012	1.095 ± 0.013	1.099 ± 0.013	1.101 ± 0.014	1.101 ± 0.015
**Static SRR**	1.081 ± 0.012	1.088 ± 0.015	1.096 ± 0.017	1.094 ± 0.017	1.096 ± 0.017	1.100 ± 0.017
**RG-BFFE-MI**	1.022 ± 0.023	1.027 ± 0.031	1.031 ± 0.034	1.039 ± 0.037	1.043 ± 0.038	1.044 ± 0.038
**RG-SST2WAx-MI**	1.013 ± 0.004	1.015 ± 0.005	1.018 ± 0.004	1.021 ± 0.005	1.023 ± 0.004	1.026 ± 0.004
**RG-MRCP-MI**	1.009 ± 0.006	1.010 ± 0.006	1.013 ± 0.007	1.012 ± 0.006	1.012 ± 0.006	1.015 ± 0.007
**RG-SST2WAx-NMI**	–	–	–	–	–	–
**RG-MRCP-NMI**	–	–	–	–	–	–

(d) PSNR

MC Strategy	a+c	a+c+s	2a+2c+2s	a+c+s+3obl	a+c+s+4obl	2a+2a+2c+4obl
**NiftyMIC**	15.001 ± 2.926	15.135 ± 2.902	15.030 ± 2.847	15.148 ± 2.863	15.123 ± 2.850	15.061 ± 2.832
**RG-HRT2W-MI**	14.162 ± 2.938	14.252 ± 2.990	14.398 ± 3.013	14.474 ± 3.031	14.498 ± 3.027	14.546 ± 3.043
**RG-BFFE-MI**	12.536 ± 2.219	13.328 ± 2.339	13.903 ± 2.337	14.412 ± 2.505	14.644 ± 2.541	14.904 ± 2.513
**RG-BFFE-NCC**	14.302 ± 3.043	14.257 ± 3.034	14.298 ± 3.020	14.378 ± 3.042	14.364 ± 3.025	14.365 ± 3.019
**RG-HRT2W-NCC**	14.275 ± 3.048	14.230 ± 3.027	14.276 ± 3.011	14.349 ± 3.042	14.344 ± 3.027	14.350 ± 3.020
**RG-BFFE-NMI**	14.243 ± 3.062	14.219 ± 3.048	14.244 ± 3.026	14.330 ± 3.051	14.313 ± 3.034	14.305 ± 3.025
**RG-HRT2W-NMI**	14.148 ± 3.049	14.129 ± 3.025	14.164 ± 3.007	14.251 ± 3.029	14.236 ± 3.011	14.232 ± 3.003
**RG-MRCP-NCC**	13.968 ± 2.803	13.984 ± 2.849	14.039 ± 2.850	14.151 ± 2.882	14.148 ± 2.877	14.150 ± 2.875
**RG-SST2WAx-NCC**	13.929 ± 2.983	13.906 ± 2.940	13.985 ± 2.936	14.130 ± 2.975	14.159 ± 2.967	13.426 ± 2.499
**Static SRR**	13.727 ± 2.827	13.746 ± 2.794	13.957 ± 2.938	13.926 ± 2.782	13.932 ± 2.759	14.041 ± 2.886
**RG-SST2WAx-MI**	11.822 ± 1.401	12.659 ± 1.686	13.437 ± 1.829	13.852 ± 1.976	14.201 ± 2.073	14.565 ± 2.158
**RG-MRCP-MI**	11.754 ± 1.369	12.658 ± 1.608	13.596 ± 1.824	13.529 ± 1.873	13.752 ± 1.915	14.239 ± 1.967
**RG-SST2WAx-NMI**	–	–	–	–	–	–
**RG-MRCP-NMI**	–	–	–	–	–	–

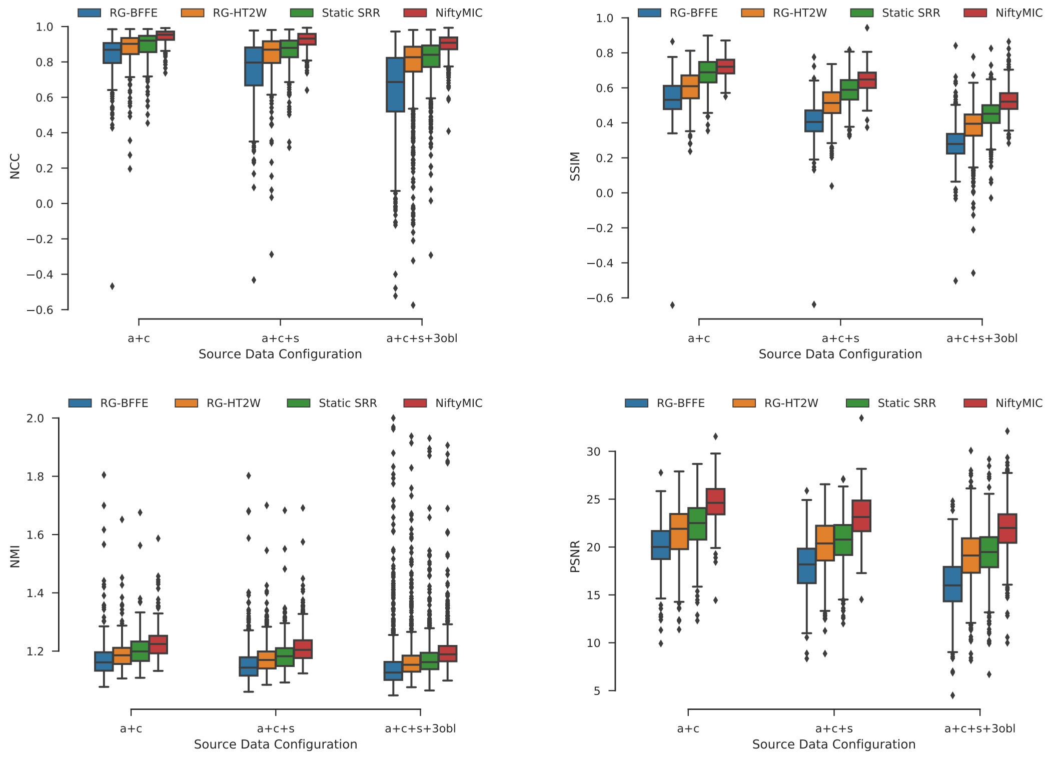

FIGURE S5 Projected slice similarity evaluation for all slices of obtained abdominal SRRs for an increasing number of input stacks for different motion correction strategies summarized for all eight subjects.

TABLE S2 Projected slice similarity evaluation of obtained abdominal SRRs for an increasing number of input stacks for different motion correction strategies summarized for all eight subjects. The rows are sorted in a descending order according to the NCC/NMI-outcome for "a+c+s+3obl". NiftyMIC shows superior self-consistency across different number of input data scenarios.

(a) NCC and SSIM

	NCC			SSIM		
**MC Strategy**	**a+c**	**a+c+s**	**a+c+s+3obl**	**a+c**	**a+c+s**	**a+c+s+3obl**
**NiftyMIC**	0.942 ± 0.041	0.922 ± 0.050	0.898 ± 0.061	0.720 ± 0.056	0.645 ± 0.066	0.526 ± 0.072
**Static SRR**	0.892 ± 0.084	0.858 ± 0.093	0.812 ± 0.129	0.680 ± 0.089	0.584 ± 0.086	0.447 ± 0.085
**RG-HT2W-NCC (RigidOnly)**	0.872 ± 0.120	0.835 ± 0.144	0.778 ± 0.193	0.602 ± 0.109	0.511 ± 0.098	0.390 ± 0.104
**RG-HT2W-NCC**	0.871 ± 0.110	0.835 ± 0.138	0.772 ± 0.201	0.599 ± 0.102	0.506 ± 0.095	0.381 ± 0.107
**RG-BFFE-NCC (RigidOnly)**	0.834 ± 0.114	0.751 ± 0.172	0.651 ± 0.226	0.541 ± 0.100	0.414 ± 0.094	0.285 ± 0.095
**RG-BFFE-NCC**	0.832 ± 0.136	0.751 ± 0.179	0.648 ± 0.229	0.537 ± 0.122	0.409 ± 0.108	0.282 ± 0.098
**RG-HT2W-MI**	0.748 ± 0.158	0.687 ± 0.193	0.577 ± 0.248	0.500 ± 0.151	0.385 ± 0.115	0.257 ± 0.103
**RG-HT2W-MI (RigidOnly)**	0.749 ± 0.151	0.678 ± 0.171	0.565 ± 0.218	0.498 ± 0.146	0.372 ± 0.103	0.247 ± 0.092
**RG-BFFE-MI**	0.713 ± 0.188	0.634 ± 0.208	0.509 ± 0.233	0.446 ± 0.136	0.333 ± 0.108	0.215 ± 0.092
**RG-BFFE-MI (RigidOnly)**	0.706 ± 0.190	0.627 ± 0.201	0.505 ± 0.216	0.441 ± 0.138	0.334 ± 0.102	0.217 ± 0.085
**RG-HT2W-NMI**	–	–	–	–	–	–
**RG-HT2W-NMI (RigidOnly)**	–	–	–	–	–	–
**RG-BFFE-NMI**	–	–	–	–	–	–
**RG-BFFE-NMI (RigidOnly)**	–	–	–	–	–	–

(b) NMI and PSNR

	NMI			PSNR		
**MC Strategy**	**a+c**	**a+c+s**	**a+c+s+3obl**	**a+c**	**a+c+s**	**a+c+s+3obl**
**NiftyMIC**	1.230 ± 0.058	1.215 ± 0.061	1.203 ± 0.076	24.631 ± 2.170	23.217 ± 2.251	21.903 ± 2.434
**Static SRR**	1.205 ± 0.062	1.188 ± 0.059	1.180 ± 0.083	22.229 ± 2.689	20.631 ± 2.566	19.356 ± 2.546
**RG-HT2W-NCC**	1.192 ± 0.060	1.179 ± 0.062	1.174 ± 0.092	21.493 ± 2.927	20.160 ± 2.716	18.975 ± 2.893
**RG-HT2W-NCC (RigidOnly)**	1.194 ± 0.067	1.180 ± 0.065	1.174 ± 0.089	21.617 ± 3.003	20.250 ± 2.807	19.116 ± 2.846
**RG-HT2W-MI**	1.175 ± 0.099	1.162 ± 0.100	1.162 ± 0.134	17.818 ± 3.646	16.453 ± 3.333	14.918 ± 3.360
**RG-BFFE-MI**	1.175 ± 0.103	1.168 ± 0.112	1.161 ± 0.121	17.031 ± 3.119	15.417 ± 3.086	13.680 ± 2.789
**RG-HT2W-MI (RigidOnly)**	1.181 ± 0.127	1.163 ± 0.115	1.159 ± 0.140	17.721 ± 3.613	16.169 ± 3.246	14.530 ± 3.180
**RG-BFFE-MI (RigidOnly)**	1.171 ± 0.094	1.168 ± 0.108	1.159 ± 0.119	16.940 ± 2.924	15.269 ± 2.560	13.496 ± 2.517
**RG-BFFE-NCC**	1.181 ± 0.087	1.162 ± 0.083	1.156 ± 0.111	20.036 ± 2.583	18.020 ± 2.694	16.107 ± 2.751
**RG-BFFE-NCC (RigidOnly)**	1.181 ± 0.087	1.163 ± 0.086	1.156 ± 0.111	20.129 ± 2.436	18.090 ± 2.555	16.216 ± 2.649
**RG-HT2W-NMI**	–	–	–	–	–	–
**RG-HT2W-NMI (RigidOnly)**	–	–	–	–	–	–
**RG-BFFE-NMI**	–	–	–	–	–	–
**RG-BFFE-NMI (RigidOnly)**	–	–	–	–	–	–

TABLE S3 Ground-truth (HR T2W) similarities of obtained quasi-static control brain SRRs using first-order Tikhonov (TK1) and isotropic Total Variation (TV) regularization SRR outcomes for an increasing number of input stacks for all seven subjects. The respective regularization was applied in the final reconstruction step using RG-HRT2W-NCC as the motion-correction strategy.

(a) NCC

Regularizer	a+c	a+c+s	2a+2c+2s	a+c+s+3obl	a+c+s+4obl	2a+2c+2s+4obl
**TK1**	**0.751** ± **0.046**	**0.770** ± **0.039**	**0.775** ± **0.038**	**0.779** ± **0.038**	**0.780** ± **0.038**	**0.781** ± **0.038**
**TV (α=9e-4)**	0.730 ± 0.043	0.750 ± 0.037	0.756 ± 0.036	0.761 ± 0.036	0.763 ± 0.036	0.764 ± 0.035
**TV (α=5e-4)**	0.727 ± 0.043	0.748 ± 0.037	0.755 ± 0.036	0.759 ± 0.036	0.761 ± 0.036	0.763 ± 0.035
**TV (α=1e-4)**	0.723 ± 0.043	0.745 ± 0.037	0.753 ± 0.036	0.757 ± 0.036	0.760 ± 0.036	0.762 ± 0.035

(b) SSIM

Regularizer	a+c	a+c+s	2a+2c+2s	a+c+s+3obl	a+c+s+4obl	2a+2c+2s+4obl
**TK1**	**0.368** ± **0.047**	**0.386** ± **0.051**	**0.402** ± **0.053**	**0.407** ± **0.054**	**0.411** ± **0.055**	**0.416** ± **0.055**
**TV (α=9e-4)**	0.325 ± 0.039	0.348 ± 0.041	0.360 ± 0.044	0.363 ± 0.044	0.367 ± 0.045	0.371 ± 0.045
**TV (α=5e-4)**	0.320 ± 0.040	0.342 ± 0.043	0.356 ± 0.045	0.359 ± 0.045	0.363 ± 0.046	0.368 ± 0.046
**TV (α=1e-4)**	0.309 ± 0.041	0.330 ± 0.044	0.349 ± 0.046	0.351 ± 0.046	0.355 ± 0.046	0.363 ± 0.047

(c) NMI

Regularizer	a+c	a+c+s	2a+2c+2s	a+c+s+3obl	a+c+s+4obl	2a+2c+2s+4obl
**TK1**	**1.090** ± **0.014**	**1.103** ± **0.015**	**1.107** ± **0.016**	**1.110** ± **0.016**	**1.111** ± **0.016**	**1.112** ± **0.016**
**TV (α=9e-4)**	1.086 ± 0.012	1.097 ± 0.013	1.100 ± 0.014	1.103 ± 0.014	1.104 ± 0.014	1.104 ± 0.014
**TV (α=5e-4)**	1.083 ± 0.012	1.095 ± 0.013	1.098 ± 0.014	1.100 ± 0.014	1.101 ± 0.014	1.102 ± 0.014
**TV (α=1e-4)**	1.080 ± 0.011	1.091 ± 0.012	1.095 ± 0.013	1.097 ± 0.013	1.099 ± 0.013	1.100 ± 0.014

(d) PSNR

Regularizer	a+c	a+c+s	2a+2c+2s	a+c+s+3obl	a+c+s+4obl	2a+2c+2s+4obl
**TK1**	14.275 ± 3.048	14.230 ± 3.027	14.276 ± 3.011	14.349 ± 3.042	14.344 ± 3.027	14.350 ± 3.020
**TV (α=9e-4)**	**14.311** ± **2.821**	**14.379** ± **2.820**	**14.420** ± **2.808**	**14.496** ± **2.828**	**14.510** ± **2.819**	**14.523** ± **2.819**
**TV (α=5e-4)**	14.223 ± 2.799	14.306 ± 2.803	14.376 ± 2.798	14.448 ± 2.815	14.468 ± 2.809	14.493 ± 2.811
**TV (α=1e-4)**	14.114 ± 2.769	14.215 ± 2.781	14.325 ± 2.786	14.392 ± 2.799	14.420 ± 2.796	14.460 ± 2.802

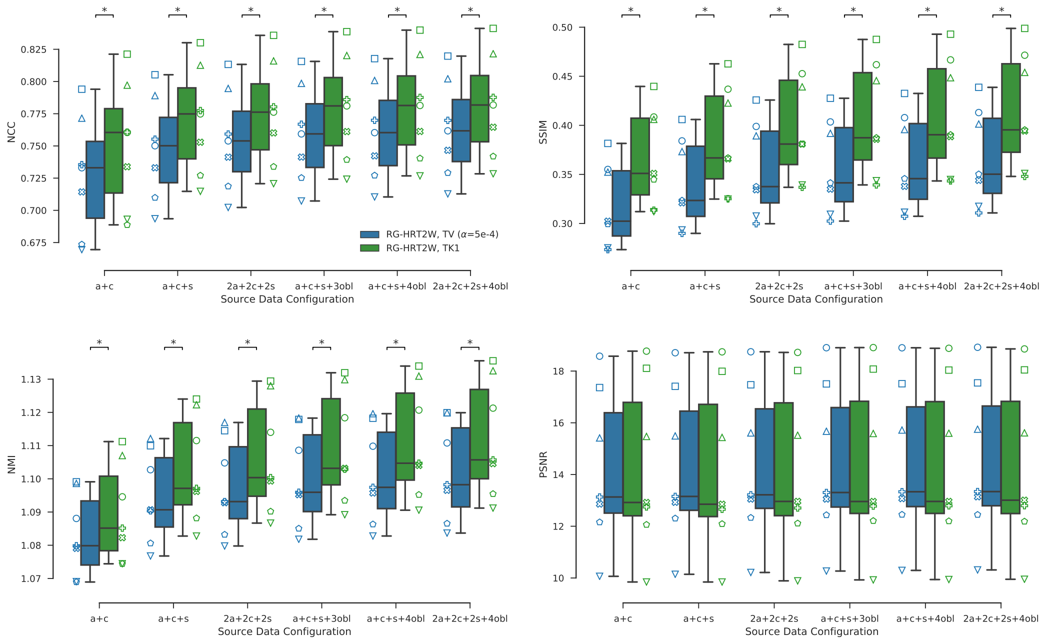

FIGURE S6 Ground-truth (HR T2W) similarities for first-order Tikhonov (TK1) and isotropic Total Variation (TV) regularization SRR outcomes for the quasi-static brain experiment involving seven subjects. The respective regularization was applied in the final reconstruction step using RG-HRT2W-NCC as the motion-correction strategy. Stars indicate statistical differences between the groups using a pairwise Wilcoxon signed-rank test ($p < 0.05$).

TABLE S4 Typical computational times to create a HR visualization of the biliary tree split into motion correction and volumetric reconstruction processing times. Motion correction for NiftyMIC refers to the total time of the two-step registration and TK1-based reconstruction iterations without the final SRR step. Volumetric reconstruction refers to solving the SRR problem (S1) after performed motion correction using either first-order Tikhonov (TK1) or isotropic total variation (TV) regularizations (S2) and (S3), respectively. The total reconstruction time is determined by the sum of the individual motion-correction and volumetric reconstruction times.

	Motion Correction		Volumetric Reconstruction	
**Source Data Configuration**	**RG-HT2W-NCC**	**NiftyMIC**	**TK1**	**TV**
**a+c**	1 min 30 sec	7 min 20 sec	1 min	17 min
**a+c+s**	2 min 30 sec	10 min 40 sec	1 min 30 sec	25 min
**a+c+s+3obl**	7 min 15 sec	32 min 15 sec	7 min 15 sec	2 h

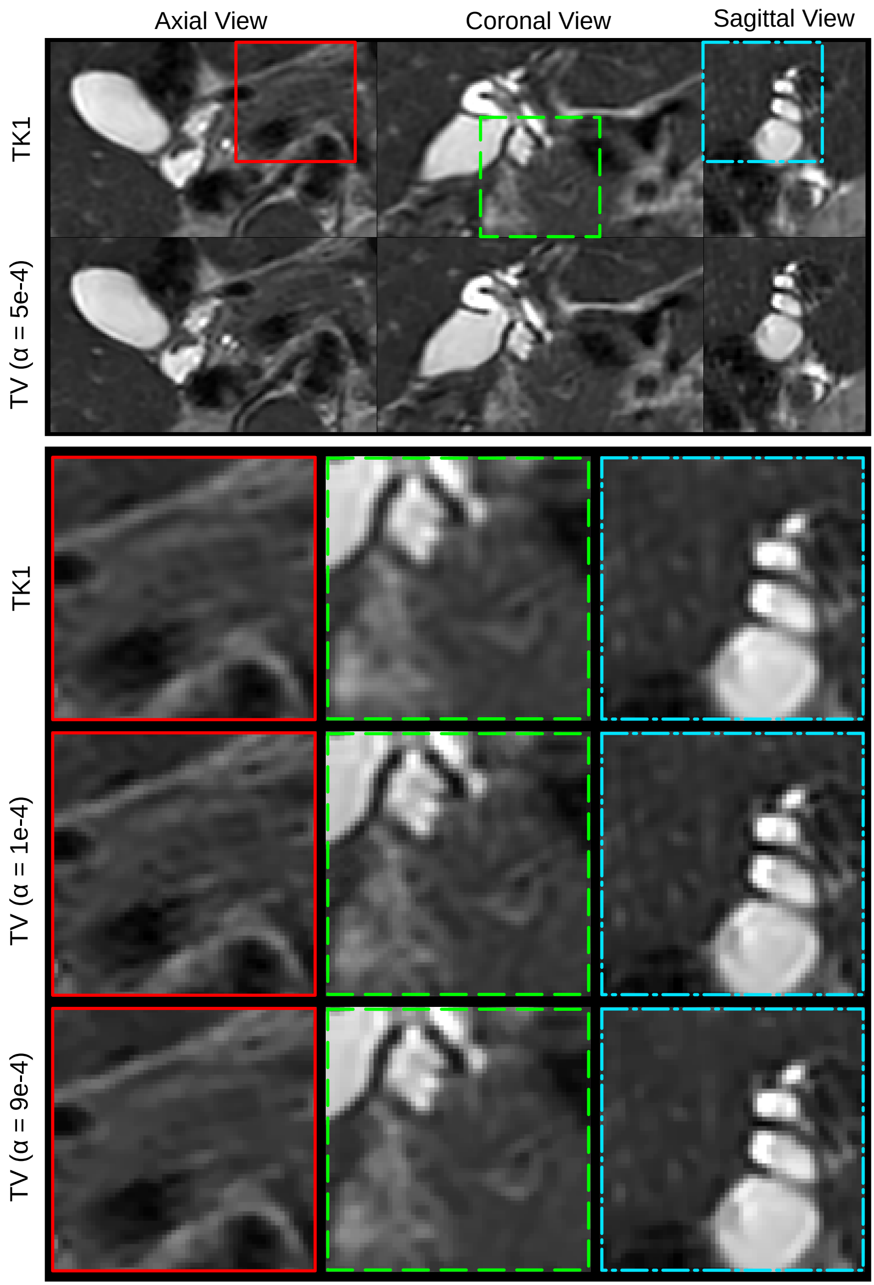

FIGURE S7 Qualitative comparison of the impact of using either first-order Tikhonov (TK1) and isotropic Total Variation (TV) regularization in the final reconstruction step using NiftyMIC (a+c+s+3obl). The zoomed windows illustrate that smaller regularization parameters α for TV result in similar SRRs as obtained by TK1. Increasing α leads to reconstructions with slightly sharper edges but at the cost of a staircasing effect typical for TV regularization [1] which presents artificial discontinuities and may suppress clinically relevant structural information.
